# Discovery of small molecule inhibitors of xyloglucan endotransglucosylase (XET) activity by high-throughput screening

**DOI:** 10.1016/j.phytochem.2015.06.016

**Published:** 2015-09

**Authors:** Dimitra Chormova, Lenka Franková, Andrew Defries, Sean R. Cutler, Stephen C. Fry

**Affiliations:** a The Edinburgh Cell Wall Group, Institute of Molecular Plant Sciences, School of Biological Sciences, The University of Edinburgh, The King’s Buildings, Max Born Crescent, Edinburgh EH9 3BF, UK; b Department of Botany and Plant Sciences, Center for Plant Cell Biology, Department of Chemistry (CFM), University of California, 5451 Boyce Hall, Riverside, CA 92521, USA

**Keywords:** IC_50_, concentration required for 50% inhibition (e.g. of XET), SR, sulphorhodamine, XET, xyloglucan endotransglucosylase (activity), XGO, xyloglucan oligosaccharide, XTH, xyloglucan endotransglucosylase/hydrolase (protein), XLLG, non-fucosylated xyloglucan nonasaccharide (Gal_2_.Xyl_3_.Glc_4_), XXFG, fucosylated xyloglucan nonasaccharide (Fuc.Gal.Xyl_3_.Glc_4_), XXXGol, reduced heptasaccharide of xyloglucan (Xyl_3_.Glc_3_.glucitol), Anthraquinones, Cell wall, Chemical genetics, Dot-blot assay, Flavonoids, Riboflavin, Singlet oxygen, Sulphydryl reagents, Tannins, Xyloglucan endotransglucosylase (XET)

## Abstract

Small molecules (xenobiotics) that inhibit cell-wall-localised enzymes are valuable for elucidating the enzymes’ biological roles. We applied a high-throughput fluorescent dot-blot screen to search for inhibitors of *Petroselinum* xyloglucan endotransglucosylase (XET) activity *in vitro*. Of 4216 xenobiotics tested, with cellulose-bound xyloglucan as donor-substrate, 18 inhibited XET activity and 18 promoted it (especially anthraquinones and flavonoids). No compounds promoted XET in quantitative assays with (cellulose-free) soluble xyloglucan as substrate, suggesting that promotion was dependent on enzyme–cellulose interactions. With cellulose-free xyloglucan as substrate, we found 22 XET-inhibitors – especially compounds that generate singlet oxygen (^1^O_2_) e.g., riboflavin (IC_50_ 29 μM), retinoic acid, eosin (IC_50_ 27 μM) and erythrosin (IC_50_ 36 μM). The riboflavin effect was light-dependent, supporting ^1^O_2_ involvement. Other inhibitors included tannins, sulphydryl reagents and triphenylmethanes. Some inhibitors (vulpinic acid and brilliant blue G) were relatively specific to XET, affecting only two or three, respectively, of nine other wall-enzyme activities tested; others [e.g. (−)-epigallocatechin gallate and riboflavin] were non-specific. *In vivo*, out of eight XET-inhibitors bioassayed, erythrosin (1 μM) inhibited cell expansion in *Rosa* and *Zea* cell-suspension cultures, and 40 μM mycophenolic acid and (−)-epigallocatechin gallate inhibited *Zea* culture growth. Our work showcases a general high-throughput strategy for discovering wall-enzyme inhibitors, some being plant growth inhibitors potentially valuable as physiological tools or herbicide leads.

## Highlights

• We screened chemical collections for XET modulators in filter-paper-based dot-blot assays.
• Some ‘hits’ inhibited, others promoted, XET activity on cellulose-bound xyloglucan.
• Most of the promoters inhibited XET when re-assayed radiochemically on soluble xyloglucan.
• The strongest XET inhibitors were singlet oxygen-generators e.g., riboflavin (IC_50_ 29 μM).
• XET inhibitors are potentially useful as tools for probing XET’s roles *in vivo*.

## Graphical abstract

Xenobiotics that inhibit the cell-wall-remodelling enzyme activity, xyloglucan endotransglucosylase (XET), would be a valuable tool in ‘chemical genetics’ for elucidating its biological roles. By screening 4216 small molecules in a dot-blot assay on paper-immobilised xyloglucan, we discovered 45 compounds that inhibited or promoted XET activity. The most effective inhibitors were singlet oxygen generators.

**Figure:**
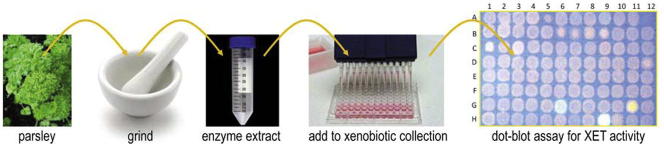

## 1 Introduction

Xyloglucan endotransglucosylase (XET), a homo-transglycanase enzyme activity found in all land plants and in some charophytic algae (Eklöf and Brumer, 2010, Fry et al., 1992, Nishitani and Tominaga, 1992, Rose et al., 2002, Vissenberg et al., 2000, Franková and Fry, 2013), acts *in vivo* during the initial assembly (Thompson et al., 1997) and subsequent re-structuring (Thompson and Fry, 2001) of the xyloglucan–cellulose network in primary cell walls. XET is one of two activities exhibited by a class of proteins known as XTHs (xyloglucan endotransglucosylase/hydrolases), 33 of which are encoded in the *Arabidopsis thaliana* genome (Nishitani, 2005, Eklöf and Brumer, 2010); the second activity is xyloglucan endohydrolase (XEH), which is the predominant activity of a minority of XTHs (Shi et al., 2015). All known plant XTHs belong to CAZy class GH16 (Nishitani, 2005, Strohmeier et al., 2004). A related hetero-transglycanase activity, MXE (mixed-linkage-glucan:xyloglucan endotransglucosylase), has been detected in *Equisetum* and certain charophytes (Fry et al., 2008). Other homo-transglycanase activities potentially acting on plant cell walls include trans-β-mannanase (Schröder et al., 2009) and trans-β-xylanase (Franková and Fry, 2011, Derba-Maceluch et al., 2014). It is likely that the various transglycanases play biologically important roles in plants (Franková and Fry, 2013). Methods for assaying diverse transglycanase activities have been reviewed and extended (Franková and Fry, 2015).

The precise roles of XET and other transglycanase activities in growth and development remain unclear. One general approach to exploring the functions of enzymes is the genetic strategy of knocking out, or altering the expression of, the genes encoding them. However, it would be difficult to knock out all 33 *A. thaliana* XTHs simultaneously, and a totally XET-deficient plant might well be embryo-lethal and thus useless for investigating the range of XET’s roles *in planta*. In principle, an alternative strategy that circumvents this obstacle is ‘chemical genetics’: treating plant cells with specific, non-phytotoxic, enzyme inhibitors (Nagahashi and Fleet, 1990, Gruner et al., 2002, Hicks and Raikhel, 2012, Nicotra et al., 2009, Winchester and Fleet, 1992). Xenobiotics that target the active site common to all XTHs could potentially be applied at any desired stage of development, and the consequences of a sudden block in XET action observed. To date, however, there are no known specific inhibitors of XET activity. We are therefore screening xenobiotic collections for inhibitors of this and other wall enzyme activities.

In some areas of plant science, a potential problem with applying ‘chemical genetics’ to inhibit enzymes is that the xenobiotics may not penetrate the plasma membrane and may thus fail to reach the (intraprotoplasmic) target enzyme. For this reason, the Lipinski “rule of 5” (Lipinski et al., 2001) may be recommended as a route to finding hits, avoiding unduly large or hydrophilic compounds for example. However, membrane impermeability is turned to an advantage in the case of apoplastic enzymes such as XTHs: a xenobiotic would not need to pass through any membranes to gain access to the target enzyme; indeed, membrane-impermeant xenobiotics would have a more specific effect since they would not be capable of reaching intraprotoplasmic enzymes that are irrelevant to the cell-wall processes of interest. Permeation of the wax and cuticle in stems and leaves of whole plants could remain a problem, but one which would not arise in studies of root or callus growth.

To begin a search for inhibitors of XET activity, we have devised a strategy for screening large numbers of xenobiotics to detect any that inhibit XET activity *in vitro*. The screen is based on the enzymes’ ability to catalyse the reaction of xyloglucan (donor substrate) with a sulphorhodamine-labelled xyloglucan-oligosaccharide (XGO–SR; acceptor substrate), within a filter-paper matrix, to generate a high-M_r_ xyloglucan–SR product which remains on the paper during a washing step and shows up as a fluorescent spot (Fry, 1997). This dot-blot screen has the advantage of handling numerous xenobiotics in a ‘single pipetting’ procedure: the xenobiotic/enzyme mixture is pipetted directly onto a test paper, which is then simply incubated, washed and recorded. In standard radiochemical assays (Fry et al., 1992), in contrast, the xenobiotic/enzyme mixture would be pipetted into a substrate solution, incubated, and again pipetted onto a paper, which is then washed, cut into rectangles and finally added to scintillation fluid. Thus the radiochemical assay is more labour-intensive. Nevertheless, it has the advantage of yielding rigorously quantitative data, and was therefore used in the present work for re-screening smaller numbers of xenobiotics provisionally identified as ‘hits’ in the dot-blot screen.

Since our goal was to discover agents that inhibit the XET activity of *all* XTHs, we screened the action of xenobiotics on a crude plant extract rather than on any specific purified XTH protein. An initial survey was therefore conducted enabling us to identify convenient sources of XET activity in which the yield of product is proportional to the concentration of the enzyme.

Xenobiotics selected for high-throughput screening included compounds related to cell-wall constituents, as well as substances known to inhibit certain glycosidases and esterases, several general enzyme inhibitors and pharmaceuticals, and the ‘LATCA’ collection [Library of AcTive Compounds on Arabidopsis] (Zhao et al., 2007; http://cutlerlab.blogspot.co.uk/2008/05/latca.html). As a result of the screening, we now report several classes of xenobiotics that modulate XET activity. We have also tested the effectiveness of a sub-set of the XET inhibitors as inhibitors of cell expansion.

## 2 Results

### 2.1 Selection of parsley as preferred source of XET activity

**Fig. 1:**
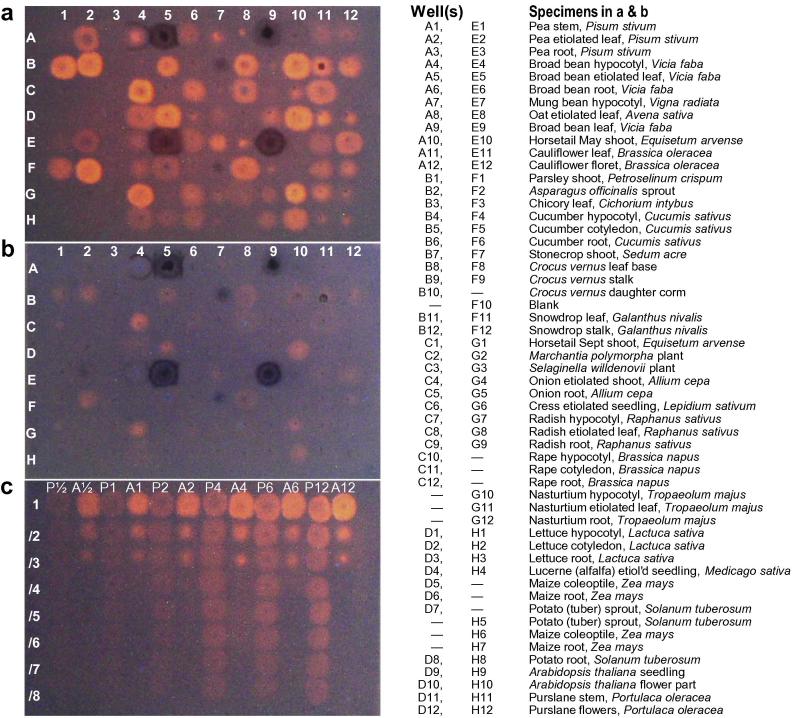
Dot-blot screening for XET activity in total extracts from diverse plant organs. (a) XET assays in 96-well format, on paper impregnated with 0.3% xyloglucan + 5 μM XGO–SR; (b) control assays on paper impregnated with XGO–SR alone. Rows A–D and E–H show results with low- and high-salt extracts, respectively, from the plant organs listed on the right. The enzyme solutions (4 μl) were incubated on the papers for 13 h at 22 °C. (c) XET assays on paper impregnated with 0.3% xyloglucan + 5 μM XGO–SR. The enzyme extracts (low-salt buffer) were from parsley (P) or asparagus (A), and either undiluted (row ‘1’) or 2–8-fold diluted (rows ‘/2’ to ‘/8’); 4 μl was applied to the paper and incubated at 22 °C for 0.5, 1, 2, 4, 6 or 12 h, as indicated.

To identify a preferred plant enzyme source for the xenobiotic survey, we tested extracts from 25 species including dicots, poalean monocots, other monocots, and non-flowering plants for XET activity using a visual ‘dot-blot’ assay (Fig. 1a and b). This work revealed several suitable sources from which to prepare crude ‘total extracts’ with high screenable XET activity, and simultaneously gave new insight into important characteristics of the ‘dot-blot’ methodology. There was considerable variability between plant organs in their extractable XET activity, but in general the presence of 1 M NaCl in the extractant (used to solubilise ionically bound enzymes) had little effect. Some extracts, e.g. from broad bean leaves (Fig. 1a; wells A5, A9, E5, E9), contained co-extracted secondary metabolites which interfered with detection of the fluorescent product, and were therefore avoided. Others showed transglycanase activity even on papers that had not been impregnated with xyloglucan as the intended donor substrate (Fig. 1b). This effect may be due to the presence of traces of soluble xyloglucan co-extracted with the enzymes. Such specimens were also avoided so that we could be confident that any observed activities were dependent on the deliberately added xyloglucan.

Some extracts gave uniform discs of the fluorescent transglycosylation product, e.g. parsley shoots (Fig. 1a; wells B1, F1), asparagus sprouts (B2, F2), etiolated onion shoots (4C, 4G), crocus corms (10B) and *Arabidopsis* flowers (10D, 10H). However, others gave sharply focused spots of fluorescent product, suggesting that the enzyme quickly bound to the paper at the point of contact with the pipette tip rather than diffusing to form a uniform disc, or that the enzyme was extracted in the form of a xyloglucan–protein or tannin–protein complex with limited ability to diffuse. Examples of extracts giving sharply focused spots include mung bean hypocotyls (7A, 7E), oat leaves (8A, 8E), purslane shoots (11D, 11H) and rape hypocotyls (10C).

To test the proportionality between signal strength (fluorescence of the spots) and the opportunity given for enzyme action (enzyme concentration × incubation time), we prepared dot-blots showing time-courses with various concentrations of the enzyme (Fig. 1c). Dilution of asparagus enzyme led to the sharply focused type of spot being formed instead of the uniform disc which had been observed at higher enzyme concentrations. Just 2–3-fold dilution was sufficient for this effect; 4-fold or greater dilution essentially abolished asparagus XET activity as detected by this method. Furthermore, the 3-fold diluted asparagus enzyme appeared to be active for only the first ∼4 h, after which time little further increase in fluorescent product occurred. In contrast, the parsley enzyme always gave a uniform disc of fluorescent product, whose intensity appeared proportional to both enzyme concentration and incubation time. The parsley extract was therefore a suitable enzyme preparation on which to test for xenobiotic inhibitors of XET activity, since the degree of inhibition would be faithfully reported by the diminished intensity of a uniform fluorescent disc.

### 2.2 Initial high-throughput screening of xenobiotics by dot-blot

**Fig. 2:**
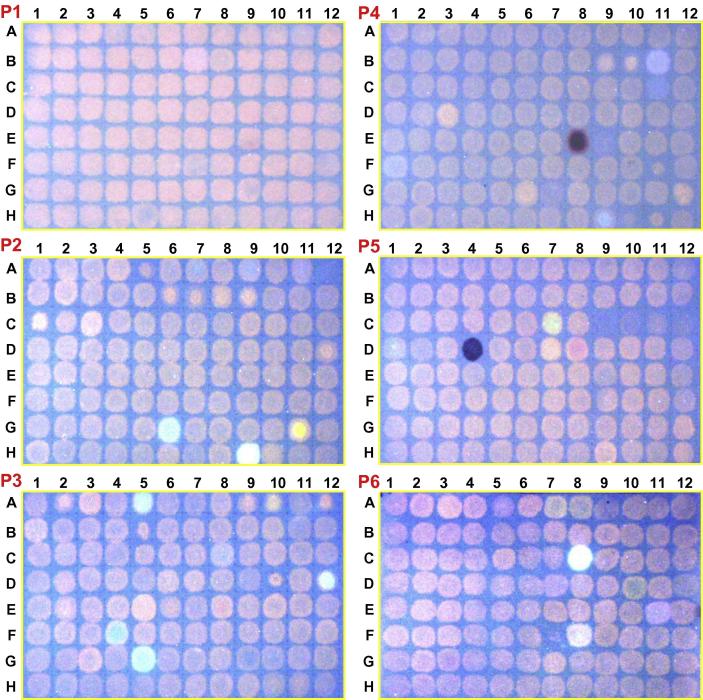
Representative dot-blot screens for inhibitors of parsley XET activity. The papers had been impregnated with 0.3% xyloglucan + generally about 5 μM XGO–SR (though the exact concentration varied, which accounts for the differences between papers in the fluorescence intensity of the XET products). Parsley enzyme extract (4 μl) containing a specific xenobiotic (200 μg/ml) was pipetted onto each station. After 2 h incubation under humid conditions, the papers were washed and fluorescent reaction products of XET activity were recorded. The results are shown here for the six plates (P1–P6) representing the EDI collection of xenobiotics.

The XET-rich parsley extract was tested on 53 dot-blot sheets, in 96-well format, in the presence of 4216 xenobiotics (plus controls), as a survey for potential inhibitors of total XET activity in a preparation containing mixed XTHs. Representative sheets are shown in Figs. 2 and S1, and the preliminary hits revealed are listed in Table 1 (‘dot-blot’ column). Compared with xenobiotic-free controls, three principal effects were noted, which are scored in Table 1 as coagulation (**c**), inhibition (**i**) and promotion (**p**). In addition, a few of the xenobiotics could not be fully washed out of the papers with our standard solution (ethanol/formic acid/water) or with any alternatives tried (acetone, DMSO, pure ethanol, dimethylformamide or aqueous sodium dodecyl sulphate), and either exhibited strong autofluorescence (e.g. riboflavin, norharmane, coumestrol, rutin and anthrone) or showed up as dark, UV-absorbing spots (e.g. bipyridyl and nigrosine), precluding observation of any XET-generated orange-fluorescing spot. Nevertheless, many other brightly coloured xenobiotics (e.g. brilliant blue G and R, toluidine blue O, bromocresol green, bromocresol purple, eosin Y, orange G and ruthenium red) were successfully washed out of the paper so that the underlying orange-fluorescing xyloglucan–SR product of XET activity could be clearly recorded.

**Table 1:** Summary of all putative ‘hits’ for effects of xenobiotics on parsley XET activity.




Plates: P1–P6 = EDI collection; plates L1–L47 = LATCA collection.

Dot-blot results (effect on parsley XET activity; see Figs. 2 and S1): 0, no effect on activity; a, not testable by dot-blot owing to autofluorescence; c, coagulation (with i, 0 or p); i, inhibition; p, promotion. ^3^H (radiochemical) XET assay results: 0, no effect; i, ii, iii, weak, moderate, strong inhibition; [blank], no data. IC_50_ = concentration causing 50% inhibition of XET activity in the radiochemical assay (see Fig. 5).

Fig.: Structure illustrated in Fig. 6 or 9.

Class (of compounds): TM, triphenylmethanes; X, xanthenes; ^1^O_2_, other singlet oxygen generators; Tan, tannins; F, flavonoids (excluding tannins); SH, sulphydryl modifiers; U, unclassified. [Some compounds are entered twice because two independent sources of these compounds were tested.]

β-Gal and β-Xyl results (inhibition of parsley β-galactosidase + galactanase and β-xylosidase + xylanase, tested on the eight XET inhibitors selected for biological testing): 0, no effect; i, ii, iii, weak, moderate, strong inhibition.

^*^Assumed, for estimation of IC_50_, to be decagalloyl glucose.

^†^These compounds are also reported on in Table 3 and Fig. 8.

The ‘type c’ effect (coagulation), produced strongly by 13 compounds, resulted in a fluorescent spot that was much smaller than in the xenobiotic-free control. Sometimes the compact fluorescent spot had an intensity about equal to that of the xenobiotic-free controls (scored as ‘c,0’); in other cases, however, it was promoted (‘c,p’) or inhibited (‘c,i’) in intensity. The typical ‘type c’ coagulation effect (caused by xenobiotics) resembled that observed when asparagus enzyme was excessively diluted (Fig. 1c). It was seen particularly with polyphenolic- or tannin-related compounds such as apigenin, phloretin, luteolin, baicalein, epigallocatechin gallate and phenolphthalein. Such astringent compounds may coagulate water-soluble enzymes such as XTHs; however, they frequently did *not* perceptibly inhibit the XET activity. No coagulation was produced by xenobiotics from the LATCA collection (which were applied at 10–40-fold lower concentrations).

The ‘type i’ effect (inhibition without coagulation) gave either a fainter fluorescent spot than in the control but of similar size, or the complete absence of a spot. This was observed with 18 compounds in the initial screen: 9 out of 566 unique to the EDI collection, 8 out of 3650 unique to the LATCA collection, and one (4-chloromercuribenzoate) that was included in both. The identities of these compounds are discussed after the results of re-testing.

Still other compounds gave an enhanced fluorescent spot, suggesting promotion of XET activity. This ‘type p’ effect (promotion of XET activity, without coagulation) was noted for 18 xenobiotics: 15 from the EDI collection and 3 from the LATCA collection. As in the case of the coagulation effect, this predominance of EDI hits was probably due to our testing of these at higher concentrations. Some were simple anions (buffered xylonic, lactobionic and phthalic acids). Another set of apparent promoters were derivatives of anthraquinone (synonyms: anthracene-9,10-dione or 9,10-dioxoanthracene) possessing at least one –OH group close to a C=O group, especially daunorubicin (Fig. 2, Plate P3, well E5) and 1-amino-4-hydroxy-2-methoxyanthracene-9,10-dione (Fig. S1, Plate L18, well E6). Three other compounds likewise possessing a fragment with an –OH group *ortho* to a phenone also slightly enhanced XET activity in the dot-blot assays, namely hesperetin, phloridzin and 4′-deoxyphloridzin (Fig. 2 and Table 1). Safranine O, forskolin, natamycin and indole 3-carbinol also appeared stimulatory. We confirmed that all the ‘type p’ compounds mentioned can be washed out of plain chromatography paper in the presence of parsley extract without leaving behind fluorescent spots.

### 2.3 Radiochemical re-screening of initial EDI hits

The dot-blot screen was performed once, its major advantage being the facility to test large numbers of compounds; but inevitably this strategy will have thrown up some false positives. Therefore, we conducted radiochemical re-screens of the initial hits.

**Fig. 3:**
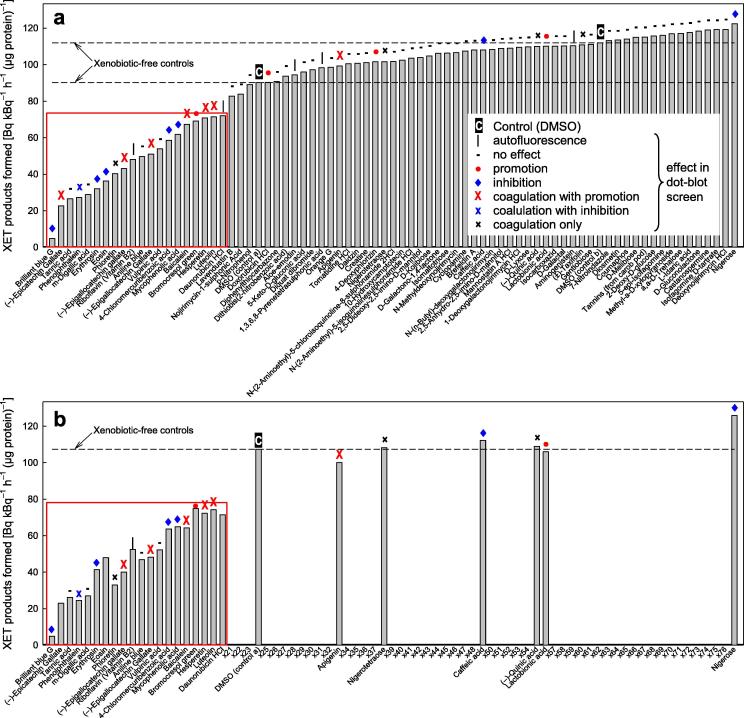
Quantitative re-testing of selected EDI collection xenobiotics for inhibition of XET activity. From the EDI collection, 27 XET dot-blot hits (Fig. 2) plus 47 chemicals that had inhibited various cell-wall glycosidase activities (data not shown) were re-screened for effects in the radiochemical XET assay. Two ‘samples’ were DMSO-only controls (dashed lines; ‘C’). The compounds’ previous behaviour in the dot-blots is summarised by symbols above the relevant bar. (a) All compounds mentioned, samples listed along the *x*-axis in order of XET activity. (b) Selected compounds for second re-testing; samples are listed in the same order as in (a), omitting those indicated by ‘x’ (x21, x22, etc.). Each value is the mean of two assays. Detailed data on the compounds tested, with cross-references to the dot-blot wells shown in Fig. 2, are given in Table S1. Red boxes highlight compounds that are appreciably inhibitory. (For interpretation of the references to colour in this figure legend, the reader is referred to the web version of this article.)

A selection of the EDI xenobiotics found to affect XET activity in dot-blots (Table 1) and, as a test for specificity, 47 additional compounds that had been identified in different assays as inhibiting various cell-wall glycosylhydrolases (data not shown) were re-tested for effects on parsley XET activity by a quantitative radiochemical assay (Fig. 3a). Detailed data, with cross-references to the dot-blots shown in Fig. 2, are listed in Table S1. Most of the xenobiotics were tested at a final concentration of 200 μg/ml (≈200–800 μM) in the XET reaction mixture. The hits are summarised in the ‘^3^H assay results’ column #1 of Table 1. In this screen, 19 xenobiotics proved inhibitory, the most effective being brilliant blue G, epicatechin gallate, tannic acid, phenolphthalein, *m*-digallic acid, erythrosin B, eosin and phloretin. Of these 19 inhibitors, 13 had shown up in the dot-blot screen as hits of various types: 7 as coagulators (of which 5 had appeared slightly promotory in the dot-blot test), 5 as non-coagulating inhibitors, and one as a non-coagulating promoter. None of the xenobiotics was appreciably stimulatory in the radiochemical assay (Fig. 3a). Of the ∼55 xenobiotics showing little effect in the radiochemical assay compared with the DMSO-only controls, only 9 had shown any perceptible effect in the dot-blot screens. Thus, the dot-blot and radiochemical screens tended to agree with each other in identifying XET ‘hits’, although the dot-blot screen sometimes scored compounds as promoters that in free solution were XET inhibitors.

A second re-screening of the initial EDI hits was conducted. The 19 xenobiotics that had appeared to be XET inhibitors in the first radiochemical screen (Fig. 3a) were re-tested in a similar experiment (Fig. 3b; qualitatively summarised in the ‘^3^H assay results’ column #2 of Tables 1 and S1). All 19 were confirmed to be inhibitory. A selection of six non-inhibitory xenobiotics was also re-tested, and confirmed to have negligible effect on XET activity (Fig. 3b). Thus the radiochemical screens were reproducible.

### 2.4 Radiochemical re-screening of initial LATCA hits

**Fig. 4:**
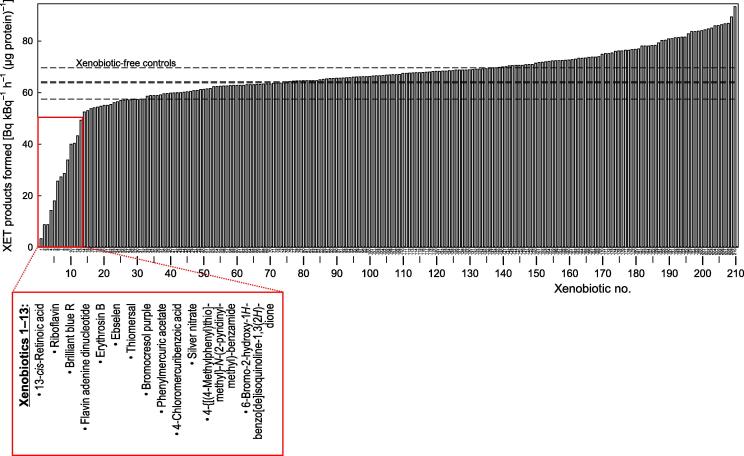
Quantitative re-testing of selected LATCA xenobiotics and some additional EDI collection xenobiotics for inhibition of XET activity. A selection of 206 compounds (mostly from the LATCA collection; plus a few additional ones from the EDI collection), most of which had shown inhibitory activity against XET (Fig. S1) and/or a cell-wall glycosidase (data not shown) in preliminary high-throughput screens, were re-screened for effects in the radiochemical XET assay. Samples are listed on the *x*-axis in order of remaining XET activity. Four ‘samples’ (Nos. 31, 74, 75 and 138) were DMSO-only controls (dashed lines). Samples 1–13, showing the strongest evidence of inhibition here, were respectively: 13-cis-retinoic acid, riboflavin, brilliant blue R, flavin adenine dinucleotide, erythrosin B, ebselen, thiomersal, bromocresol purple, phenylmercuric acetate, 4-chloromercuribenzoic acid, silver nitrate, 4-{[(4-methylphenyl)thio]methyl}-*N*-(2-pyridinylmethyl)-benzamide and 6-bromo-2-hydroxy-1*H*-benzo[de]isoquinoline-1,3(2*H*)-dione. Each value is the mean of two assays. Details of all the compounds tested, with cross-references to the relevant dot-blot wells, are given in Table S1. Red box highlights compounds that are appreciably inhibitory. (For interpretation of the references to colour in this figure legend, the reader is referred to the web version of this article.)

A selection of 206 compounds (mostly from the LATCA collection), most of which had shown inhibitory activity against either certain glycosylhydrolases (data not shown) or in a few cases XET (Figs. 2 and S1) in high-throughput dot-blots, were screened for effects in the radiochemical XET assay (Fig. 4; ‘^3^H assay results’ column #3 of Table 1). Detailed data, with cross-references to the dot-blot wells shown in Figs. 2 and S1, are given in Table S1. Thirteen of these 206 xenobiotics showed inhibition — in order of decreasing effectiveness: 13-*cis*-retinoic acid, riboflavin, brilliant blue R, flavin adenine dinucleotide, erythrosin B, ebselen, thiomersal, bromocresol purple, phenylmercuric acetate, 4-chloromercuribenzoic acid, silver nitrate, and 4-{[(4-methylphenyl)thio]methyl}-*N*-(2-pyridinylmethyl)-benzamide and 6-bromo-2-hydroxy-1*H*-benzo[de]isoquinoline-1,3(2*H*)-dione.

In a separate experiment, seven additional LATCA compounds, including three that had been scored ‘p’ (promotory) and four that had been scored ‘i’ (inhibitory) in the dot-blot screen, were also re-tested radiochemically at 200 μM. In some cases, moderate inhibition was demonstrated (Fig. S2; ‘^3^H assay results’ column #4 of Table 1). The most effective were two cancerostatic agents [1-amino-4-hydroxy-2-methoxyanthracene-9,10-dione (29% inhibition) and *N*′-(1*E*)-(5-bromofuran-2-yl)methylidene-2-(3-methylphenoxy)acetohydrazide (24% inhibition)], and epibrassinolide (22% inhibition).

### 2.5 Potency (IC_50_) of selected inhibitors

**Fig. 5:**
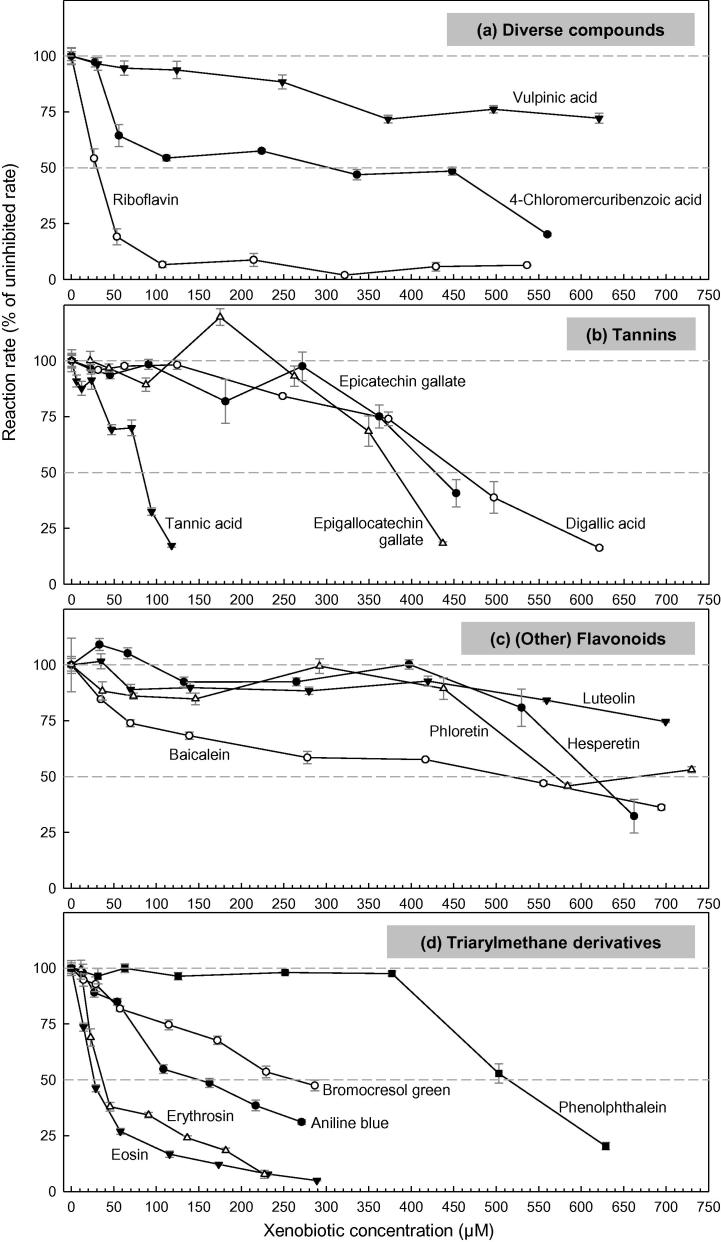
Potency of selected XET hits. Sixteen xenobiotics, confirmed as inhibitors of XET activity (Fig. 3, Fig. 4), were assayed at various concentrations in the radiochemical assay. Error bars show ±SE (*n* = 3).

**Fig. 6:**
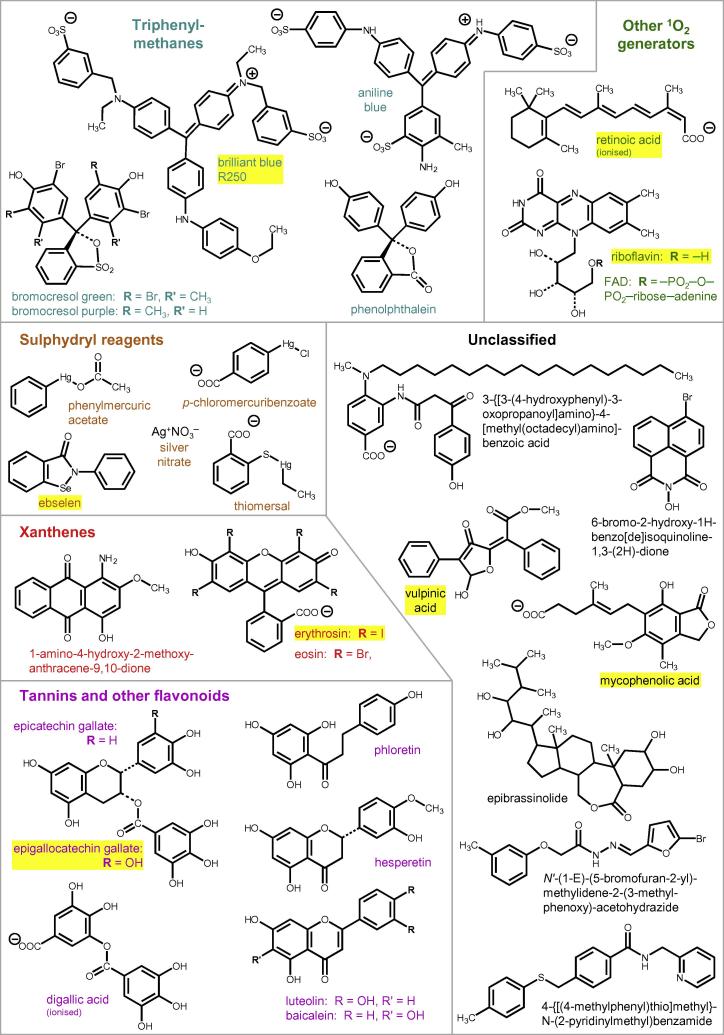
Chemical structures of the main xenobiotics that inhibited XET activity in radiochemical assays. Of the 30 compounds illustrated, 20 had previously shown up in the dot-blot tests as inhibitors, promoters and/or coagulants, and nine had had no effect; one (riboflavin) had been untestable in the dot-blot owing to autofluorescence.

A classified list of all xenobiotics observed to affect XET activity in any of the assays is given in Table 1. Sixteen of those that proved effective in radiochemical assays were tested at a range of concentrations (Fig. 5), and the IC_50_ values (concentration required for 50% inhibition of XET activity) are listed in Table 1. Representative structures are shown in Fig. 6. The most effective inhibitors (IC_50_ ≈ 30 μM) were riboflavin and two xanthene compounds (eosin Y and erythrosin B), all of which are light-dependent generators of singlet oxygen (^1^O_2_). Less effective but still inhibitory (IC_50_ 150–520 μM) were the triarylmethanes (aniline blue, bromocresol green and phenolphthalein), which are also potential ^1^O_2_ generators.

The flavonoids were moderately effective as XET inhibitors, having IC_50_ values ranging from 500 to over 700 μM. The galloyl-esterified flavonoids, conventionally regarded as tannins (epigallocatechin gallate and epicatechin gallate) were slightly more effective (IC_50_ ≈ 400 μM), and non-flavonoid gallotannins were also effective. Expressed on a molar scale, the most effective tannin-type inhibitor was tannic acid. The sulphydryl reagent, 4-chloromercuribenzoic acid, showed an unusual dose–response curve, 50–60% of the XET activity being inhibited by ∼50 μM but with little further inhibition up to ∼400 μM (Fig. 5a).

### 2.6 Influence of light on the efficacy of riboflavin

Since the effect of riboflavin proved variable in different experiments (Fig. 3, Fig. 4, Fig. 5), we suspected that its interaction with light was a determining factor. The generation of ^1^O_2_ by riboflavin is light-dependent (Choe et al., 2005). We confirmed that riboflavin strongly inhibits XET activity in the light, but only moderately in the dark (Table 2). This observation indicates that riboflavin, and probably also other compounds, can inhibit XET activity by producing singlet oxygen.

**Table 2:** Effect of 0.5 mM riboflavin and/or light on the XET reaction in solution.

Reaction mixture	[^3^H]Xyloglucan formed (cpm)	
	Low light	High light
Enzyme + substrates	8861 ± 105	8942 ± 349
Enzyme + substrates + riboflavin	5390 ± 130	807 ± 63

Parsley enzyme extract was incubated with substrates (xyloglucan and [^3^H]XXXGol) in extremely low or ‘bright indoor’ light intensity (0.2 or 1100 lux, respectively), in the presence or absence of 0.5 mM riboflavin as a singlet oxygen generator. The yield of product ([^3^H]xyloglucan) was assayed after 2 h. Data are mean ± SE of 4 independent assays.

### 2.7 Specificity of eight selected XET inhibitors tested on other wall enzymes

**Fig. 7:**
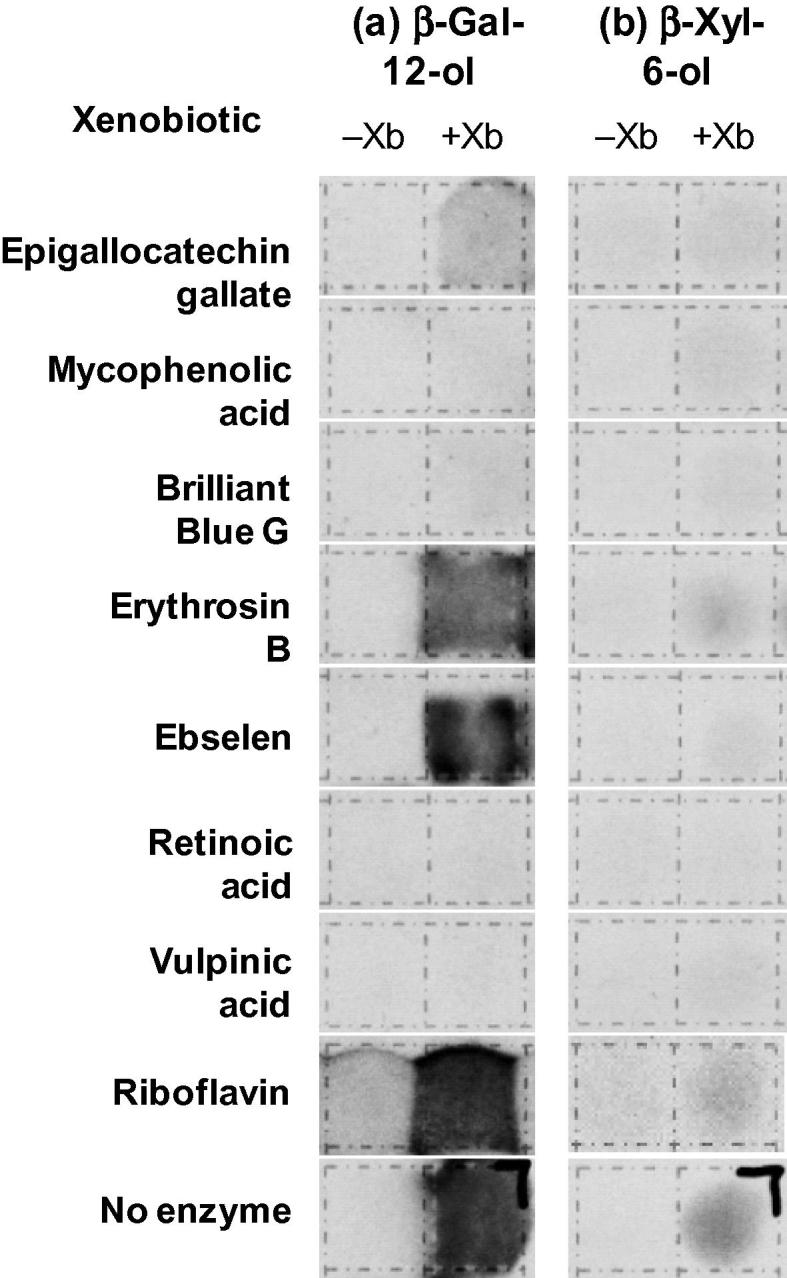
Testing the specificity of selected XET hits: dot-blot screens for inhibitors of parsley β-galactosidase/β-galactanase and β-xylosidase/β-xylanase activity. Parsley enzyme extract (5 μl) containing the named xenobiotic (200 mg/l) was incubated with (a) [1-^3^H]Gal_12_-ol or (b) [1-^3^H]Xyl_6_-ol for 24 or 48 h respectively, then the reaction mixture was dried onto plain Whatman No. 1 paper. The paper was washed with 60% ethanol (for Xyl_6_-ol) or 70% ethanol (for Gal_12_-ol), which removed the low-M_r_ hydrolysis products, then the paper was fluorographed, revealing any remaining non-hydrolysed ^3^H-oligosaccharide. The right-hand blot of each pair shows the effect of the named xenobiotic; the left-hand blot shows a control without the xenobiotic. Black spots indicate inhibition of the enzyme. In the ‘no enzyme’ sample, the right-hand blot received buffer without enzyme, mimicking the effect of a xenobiotic that completely inhibited the enzyme.

Eight xenobiotics that inhibited parsley XET activity were tested for specificity based on their ability to inhibit nine other wall-related enzyme activities. The parsley extract provided a good source of enzymes that hydrolysed Gal_12_-ol (β-galactosidase and/or β-galactanase) and Xyl_6_-ol (β-xylosidase and/or β-xylanase). Some XET inhibitors also inhibited these activities, whereas others did not (Fig. 7). Other activities were more easily detected in a lucerne (alfalfa) extract (data summarised in Table 3). No xenobiotic was found that inhibited *only* XET activity (Table 3). The most specific XET-inhibitors were vulpinic acid (a secondary metabolite of the lichen *Letharia columbiana*) and brilliant blue G, which inhibited only two and three of the other enzyme activities respectively. The broadest-spectrum inhibitors were (−)-epigallocatechin gallate and riboflavin, each of which inhibited eight of the nine other enzyme activities screened.

**Table 3:** Effect of eight selected XET inhibitors⁎ on nine other cell-wall enzyme activities from parsley and lucerne (alfalfa).

^†^See Fig. 7.

^‡^‘–’ indicates that no inhibition was observed at any concentration tested (for concentrations tested, see Fig. 8).

^¶^The compounds were tested *in vitro* on wall enzymes at 200 mg/l, which corresponds to the micromolarities listed here.

^§^i, ii, iii = weak, moderate and strong inhibition.

⁎ Structures are illustrated in Fig. 6.

### 2.8 Effect of eight selected XET inhibitors on vitality and cell expansion in cell-suspension cultures

**Fig. 8:**
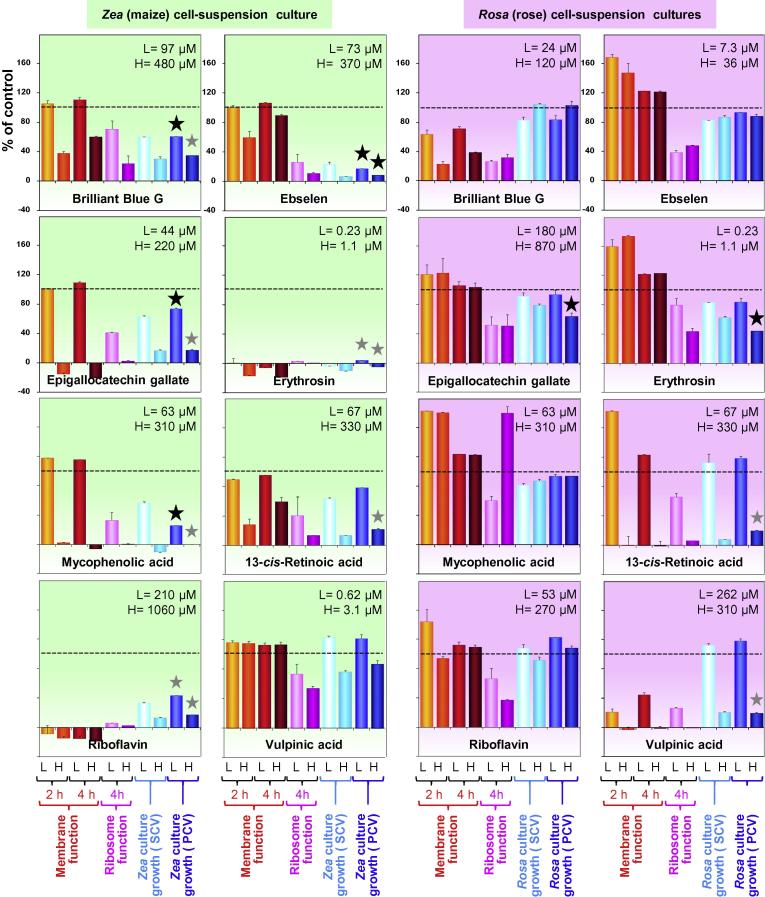
Summary of the effects of eight selected XET hits on cell-culture growth, membrane function and protein synthesis in a monocot and a dicot. Each xenobiotic was tested at two concentrations (L and H, as defined on individual histograms) for effects on membrane function (cells’ ability to take up exogenous [^14^C]proline during a 2- or 4-h interval starting 4 d after addition of the xenobiotic), ribosome function (cells’ ability to incorporate exogenous [^14^C]proline into protein during a 4-h interval starting 4 d after addition of the xenobiotic), and culture growth [assessed by the increase in packed cell volume (PCV) and settled cell volume (SCV) after 4 days’ incubation in the presence of xenobiotic]. In each case, the measurement (growth, uptake or incorporation) in the absence of xenobiotic was taken as 100%. Large black stars highlight inhibition of growth without concurrent inhibition of membrane function; small grey stars show inhibition of growth with concurrent inhibition of membrane function.

Before testing the effects of XET-inhibitors on cell expansion, we defined their non-toxic concentrations. The eight XET-inhibiting xenobiotics mentioned in §2.7 were tested for cytotoxicity in cultured *Rosa* and *Zea* cells, by simple tests of the compounds’ ability to inhibit membrane and ribosome function based on [^14^C]proline uptake and incorporation. In xenobiotic-free control 4-d-old *Rosa* cultures, 93.3% and 94.3% of the supplied [^14^C]proline was absorbed from the medium within 2 and 4 h respectively, indicating healthy membrane function. In xenobiotic-free 4-d-old *Zea* cultures, the corresponding figures were 93.9% and 92.5%. By 4 h, 5.5% of the total [^14^C]proline supplied had been incorporated into protein by *Rosa* and 9.3% by *Zea* cells, indicating healthy ribosome function. Against these control rates, we recorded any inhibition of membrane and ribosome function by the xenobiotics (Fig. 8). Each xenobiotic was tested at two concentrations (¼ and ^1^/_20_ of the lowest non-tolerated concentration), which had been selected on the basis of preliminary experiments with a dilution series of the xenobiotic.

In the *Rosa* cultures, membrane function (assessed 4 d after the xenobiotics were administered) was unaffected by most of the xenobiotics at the concentrations tested, though it was impaired by vulpinic acid and by the higher tested concentrations of brilliant blue G and retinoic acid. The *Zea* culture was generally more vulnerable than *Rosa*, its membrane function being impaired by erythrosin and riboflavin and by the higher tested concentration of brilliant blue G, epigalocatechin gallate and mycophenolic acid (though, unlike *Rosa*, not by vulpinic acid and only slightly by retinoic acid).

Ribosome function (assessed 4 d after xenobiotic application) was more often inhibited than membrane function — in part because [^14^C]protein synthesis is downstream of [^14^C]proline uptake. As with membrane function, *Rosa* protein synthesis was more resistant than *Zea*.

Cell expansion in the suspension-cultures (assessed by SCV and PCV) was strongly inhibited by several of the xenobiotics (indicated for the PCV data in Fig. 8 by a star). In some cases, this growth effect could not be attributed to any specific effect on cell expansion because the xenobiotic had proved cytotoxic (small grey star). However, with some xenobiotics this was not the case. Growth-inhibitory effects in the *absence* of membrane impairment is indicated in Fig. 8 by a large black star. These non-cytotoxic growth inhibitors were: in *Zea*, 100 μM brilliant blue G, 70 μM ebselen, 60 μM mycophenolic acid and possibly also 40 μM epigallocatechin gallate; and in *Rosa*, 1.1 μM erythrosin and 870 μM epigallocatechin gallate.

In all cases of growth inhibition, [^14^C]protein production was also inhibited. However, since protein synthesis was assessed only during the final 4 h of the 4-d period of incubation in the presence of the xenobiotic, whereas the increase in cell volume was gradually accrued over the full 4 d, it is likely that the growth effect was not simply a consequence of reduced ribosome function.

## 3 Discussion

### 3.1 Comparison of screens

The dot-blot screen, using paper-immobilised xyloglucan as donor substrate, proved simple and effective, allowing us to test over 4000 xenobiotics, manually, in about 10 days. Most of these compounds had no effect on the production of a fluorescent spot by an XET-active parsley extract, but some demonstrated clear inhibition. For example, 4-chloromercuribenzoate was included in both xenobiotic collections, and in both cases was identified as a strong inhibitor [EDI plate P2, well A12 (Fig. 2); LATCA plate L3, well C8 (Fig. S1)]. Other compounds containing mercury or silver also showed up as inhibitors (phenylmercuric acetate and AgNO_3_) in dot-blot assays. Heavy metals are well-known non-specific enzyme inhibitors, and these results offer convincing proof of concept.

The Hg and Ag compounds also reliably showed up as inhibitors when re-screened by the quantitative radiochemical XET assay using soluble xyloglucan in the absence of filter paper. Nine other compounds were also detected as inhibitors in both screens (Table 1), demonstrating a general agreement between the dot-blot and radiochemical methods.

**Fig. 9:**
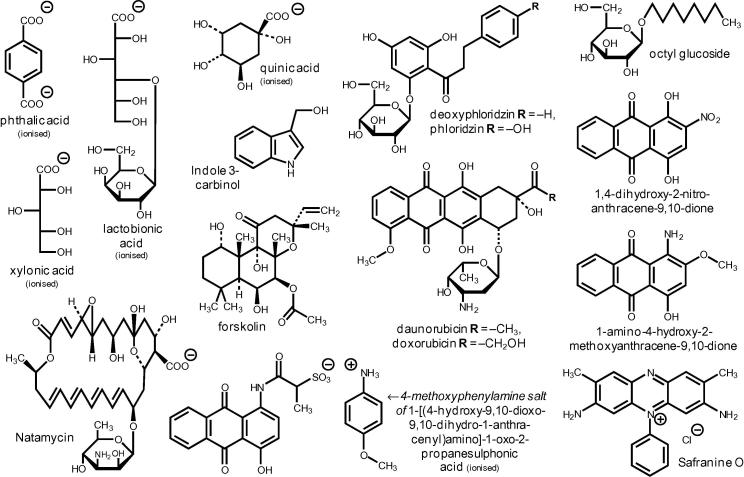
Chemical structures of xenobiotics that promoted XET activity in dot-blot assays. Of the sixteen compounds shown, six proved to have no effect when re-tested in the radiochemical assay, and two were moderately inhibitory (1-amino-4-hydroxy-2-methoxyanthracene-9,10-dione and daunorubicin); eight were not re-tested radiochemically.

However, there were also interesting discrepancies between the two screens: 11 compounds (including thiomersal, retinoic acid, FAD, vulpinic acid and three of the tannins) did not show inhibitory effects in the dot-blots but did so in radiochemical assays. Furthermore, several compounds appeared to *promote* XET activity in dot-blots (including some that were also coagulants), but had little effect or were inhibitory in the radiochemical assay (Table 1). One of the clearest examples of this was 1-amino-4-hydroxy-2-methoxyanthracene-9,10-dione (Plate L18, well E6; Figs. S1 and S2). The structures of xenobiotics that promoted XET activity in dot-blot assays are shown in Fig. 9.

There are two important differences between the two screens: in dot-blots the XET reaction was performed in the dark and within a filter-paper matrix (with cellulose-bound xyloglucan as donor substrate), whereas the radiochemical assays were conducted under normal laboratory fluorescent lighting and with the donor xyloglucan present in free solution. Both these differences may influence the observed results, for reasons discussed later.

### 3.2 Sulphydryl reagents

Hg^2+^ and Ag^+^ salts were known to inhibit XET activity in pea stem extracts (Fry et al., 1992). Hg and Ag compounds such as 4-chloromercuribenzoate, phenylmercuric acetate and AgNO_3_ are likely to act by modifying cysteine –SH or cystine –S–S– groups. Ebselen, an organic selenium compound that showed up as an XET inhibitor in both screens, is known for its ability to form Se–S bonds to cysteine residues, thereby inhibiting certain enzymes such as indoleamine-2,3-dioxygenase (Mishra et al., 2006, Terentis et al., 2009). XTHs typically possess two pairs of conserved Cys residues, which are proposed to participate in disulphide bond formation (Campbell and Braam, 1999). Reductive cleavage of the disulphide bond(s) of XTHs with 500 mM dithiothreitol can inhibit XET activity (Van Sandt et al., 2006), although another thiol reductant, 2–20 mM mercaptoethanol, was somewhat stimulatory (Fry et al., 1992).

4-Chloromercuribenzoate showed an unusual dose–response curve with almost half the XET activity being inhibited over a wide range of concentrations (50–400 μM). This implies that there are two classes of XTH, differing in the accessibility of essential cysteine or cystine residues, or that certain readily accessible Cys residue(s) are beneficial but not essential for XET activity.

### 3.3 Light-dependent generators of singlet oxygen

Riboflavin, FAD (which contains a riboflavin moiety) and 13-*cis*-retinoic acid all showed up as XET inhibitors in the radiochemical assays but not in the dot-blots (Table 1). This apparent discrepancy probably occurred because only the radiochemical screen was performed in the light, these three intensely coloured compounds being noted for their ability to generate singlet oxygen (^1^O_2_) in the light. This reactive oxygen species exerts photodynamic effects on organic molecules, including enzymes. In particular, it oxidises methionine, cysteine, histidine, tyrosine and tryptophan residues (Michaeli and Feitelson, 1995, Min and Boff, 2002). During the dot-blots, performed in the dark, little or no ^1^O_2_ would be generated, and XET activity was unaffected. Indeed, the light-dependency of riboflavin’s effect was clearly demonstrated in separate radiochemical assays (Table 2).

Other coloured compounds believed to generate ^1^O_2_ in the light include triarylmethanes (Brown et al., 2001, Buer et al., 2007, Peer and Murphy, 2007, Brezová et al., 2004). Two such compounds, erythrosin B (at the lower concentration employed in the LATCA collection) and aniline blue, resembled riboflavin in inhibiting XET activity only in the radiochemical assays, conducted in the light. Vulpinic acid similarly showed up as an inhibitor in the radiochemical assay only, and we suggest that it may also be a light-dependent ^1^O_2_-generator.

### 3.4 XET inhibitors that may operate partly via ^1^O_2_ generation

Six additional intensely coloured triarylmethanes that are potential ^1^O_2_ generators, however, did show up in both types of screen: eosin, phenolphthalein, brilliant blue R and G, bromocresol purple and bromocresol green. This behaviour indicates that these triarylmethanes can inhibit XET activity in their own right, regardless of any light-dependent ^1^O_2_ generation. They were moderately effective, having IC_50_ values of 150–500 μM (Table 1). One possible mechanism of inhibition that does not involve ^1^O_2_ is as a ‘promiscuous inhibitor’ — a compound that itself forms multimolecular aggregates (McGovern et al., 2002) capable of adsorbing and unfolding diverse enzymes (Coan et al., 2009). Certain triarylmethanes such as tetraiodophenolphthalein (which is related to erythrosin B; Fig. 6) are among the known promiscuous inhibitors (McGovern et al., 2002). However, many of our XET ‘hits’ did not inhibit the majority of other cell-wall enzymes tested, including several glycosidase activities (Table 3), so we suggest that in most cases their mode of action on XET was not as promiscuous inhibitors. In addition, erythrosin B was scored as a hit in dot-blots but only in the EDI collection (tested at higher concentrations); thus, the ^1^O_2_-independent effect of erythrosin B may be relatively weak, whereas its ^1^O_2_-dependent effect seen in the light is moderately potent (IC_50_ = 36 μM; Fig. 5d).

### 3.5 Flavonoids

Simple flavonoids were moderately effective XET inhibitors (IC_50_ ≈ 500–700 μM), as determined in the radiochemical assay. Interestingly, these compounds often exerted a stimulation of XET activity in the dot-blot screen, though that effect was usually accompanied by coagulation of the enzyme. The galloyl-esterified ‘tannin’ flavonoids (epigallocatechin gallate and epicatechin gallate; moderately effective, with IC_50_ ≈ 400 μM), were slightly more inhibitory than the non-tannin flavonoids in the radiochemical assay (Fig. 3), though (−)-epigallocatechin gallate was also scored as a coagulating promoter in dot-blot assays (Fig 2 and Table 1).

Flavonoids have been reported to possess numerous biological effects. They interfere in polar auxin transport by binding to a specific transporter (NPA-binding protein) (Brown et al., 2001, Buer et al., 2007, Peer and Murphy, 2007) with an IC_50_ of about 500 nM (Murphy et al., 2000), and this has been held responsible for the correlations observed between natural flavonoid accumulation and plant growth regulation, for example in roots (Hernández-Mata et al., 2010). Flavonoids also inhibit certain other enzymes, including phosphatases and kinases, sometimes with great potency. An early-discovered example was flavopiridol, which inhibits a cyclic-AMP-dependent protein kinase, PKA, with IC_50_ = 145 μM (Losiewicz et al., 1994). Even more potent examples were later discovered; for instance, flavopiridol inhibits CDC2 (a cyclin-dependent protein kinase) with IC_50_ = 0.4 μM (De Azevedo et al., 1996), and quercetagetin (3,3′,4′,5,6,7-hexahydroxyflavone) inhibits PIM1 (an oncogene protein kinase) with IC_50_ = 0.34 μM (Holder et al., 2007). Some flavonoids also inhibit photosynthetic enzymes such as phosphoenolpyruvate carboxylase and NADP-dependent malic enzyme at 0.2–1.5 μM (Pairoba et al., 1996). In addition, certain flavonols have been reported to be ‘promiscuous inhibitors’ (McGovern et al., 2002).

It appears from our work that flavonoids could affect plant growth via effects on XTHs. The IC_50_ is high compared with IC_50_ values reported in protein kinase studies, but our initial hits suggest that more potent flavonoids may exist.

Tannins have been reported as specific blockers of gibberellin-promoted growth, while having little effect on auxin-induced growth or on the growth of hormone-untreated plants (Corcoran et al., 1972, Green and Corcoran, 1975). This tannin effect was overcome by 10-fold elevation of the gibberellin concentration. It is possible that tannins exert this effect via their inhibitory influence on XET activity (Table 1), with additional gibberellin concentration increasing XTH synthesis (Potter and Fry, 1994).

### 3.6 Other inhibitors

Other potential inhibitory hits included mycophenolic acid (an immunosuppressant drug), and *N*′-(1*E*)-(5-bromofuran-2-yl)methylidene-2-(3-methylphenoxy)acetohydrazide, 4-bromo-*N*-[(2,3,4-trimethoxyphenyl)methyl]aniline, 2-(4-nitro-2-thienyl)-2,3-dihydro-1H-benzo[d]imidazole and 3-{[3-(4-hydroxyphenyl)-3-oxopropanoyl]amino}-4-[methyl(octadecyl)amino]benzoic acid (all pharmaceuticals). The mode of action of these xenobiotics is unknown.

### 3.7 Stimulation of XET activity by certain xenobiotics

None of the xenobiotics was appreciably stimulatory in the radiochemical assays (Fig. 3). It is therefore interesting that some compounds promoted XET activity in the dot-blot assays but had little effect (e.g. apigenin, 4′-deoxyphloridzin, lactobionic acid and quinic acid) or inhibited it (e.g. epigallocatechin gallate, baicalein, luteolin, phloretin and daunorubicin) in radiochemical assays. We propose that in most cases the difference is probably connected with the fact that in the dot-blot assays the reaction occurred within a filter-paper (cellulose) matrix whereas in the radiochemical assays it took place in free solution. Hypotheses that might account for the ability of a xenobiotic to promote XET activity on cellulose-immobilised substrates include the following.

(a) Some of the xenobiotics may have acted to enhance XET activity in the same manner as do certain anionic polysaccharides (Takeda and Fry, 2004), possibly by minimising the immobilisation of the enzyme on the cellulose fibres. XTHs tend to adsorb to many surfaces, even dialysis tubing and chromatography columns (Hrmova et al., 2007). Cellulose-binding of the XTH would decrease its diffusibility and thus its ability to come into contact with xyloglucan chains immobilised elsewhere on the same paper. A xenobiotic that prevents XTH–cellulose bonding (an effect that might be expected, for example, of the detergent *n*-octyl-β-d-glucopyranoside; Table 1), would keep the enzyme free to explore a wider area of the xyloglucan–cellulose composite for donor substrate molecules, and thus more frequently result in productive enzyme–substrate interactions, yielding a brighter fluorescent spot. Promotion of activity by low detergent concentrations has been noted previously with several enzymes (e.g. Ryan et al., 2003).
(b) Also viable is the hypothesis that some xenobiotics coagulate XTH without actually denaturing it. When pipetted onto dot-blot paper, a coagulated enzyme is less capable of spreading into a large uniform disc, instead being focused at a higher concentration close to where the pipette-tip was applied. This higher local concentration of enzyme may lead to a more intense but smaller fluorescent spot.

### 3.8 Specificity of XET inhibitors

Eight diverse xenobiotics were selected from among those that inhibited parsley XET activity, and tested for their ability to inhibit nine wall-related hydrolase activities. None of these eight xenobiotics was completely specific for XET inhibition (Table 3). The most specific XET-inhibitors were vulpinic acid and brilliant blue G. At the other extreme, (−)-epigallocatechin gallate and riboflavin inhibited the majority of hydrolase activities tested.

### 3.9 Effect of XET inhibitors on cell expansion

Several of these eight selected XET inhibitors were found to inhibit cell expansion in cultures cells of a monocot (*Zea*) and/or a dicot (*Rosa*), even at concentrations low enough not to inhibit membrane function (thus not immediately phytotoxic). The *Zea* culture was more susceptible to this selection of xenobiotics than the *Rosa* culture. Epigallocatechin gallate and erythrosine, broad-spectrum enzyme inhibitors, inhibited growth in both cultures. Compounds effective only in *Zea* were brilliant blue G, mycophenolic acid and ebselen. Other compounds from this selection of eight also inhibited growth, but with concurrent general toxicity (blocking membrane function). Based on the results to date, we cannot ascribe any growth-inhibitory effect specifically to the inhibition of XET activity. Nevertheless, the experimental strategy reported here provides excellent opportunities for future exploration of this approach towards the discovery of specific inhibitors of cell expansion.

## 4 Conclusion

This work establishes a high-throughput and convenient screen for discovering inhibitors (and potentially promoters) of the important XTH class of cell-wall enzymes. It appears likely that the dot-blot screen can not only detect inhibitors of XET activity but also reveal interesting effects of certain xenobiotics on the behaviour of XTH within a semi-solid (xyloglucan–cellulose) composite which in certain respects resembles the plant cell wall — the enzyme’s natural target. It is also possible that some of the compounds tested by us as ‘xenobiotics’ are related to substances serving natural roles in controlling the behaviour of XTHs *in vivo*.

The effective compounds identified in our screens merit future testing as experimental tools with which to investigate the enzymology of plant cell expansion. They may also reveal interesting features of the active site of XTHs, especially the high susceptibility of the enzyme to singlet oxygen generators.

These and other xenobiotics are also now available for exploration as ‘lead’ compounds, potentially delivering growth regulators that interfere in the action of XTHs, a class of enzymes vital to the cell-wall reactions involved in plant growth. Such compounds are potentially ‘botanical penicillins’, blocking the action of growth-essential plant transglycanases somewhat analogously to the way in which penicillin interferes with transpeptidase action in bacterial cell walls.

Panning for interesting new inhibitors that can be used in ‘chemical genetics’ (Zhao et al., 2007, Hicks and Raikhel, 2012) is an exciting avenue to be added to the list of tools available to plant scientists. The simple, high-throughput screens validated in the present work will facilitate progress towards that end.

## 5 Experimental

### 5.1 Extraction of XET activity from diverse plants

All procedures were performed at 4 °C. Small quantities of plant material were finely chopped and homogenised in liquid nitrogen with a pestle and mortar, then mixed with chilled extractant. Tissues available in large quantities were homogenised directly in chilled extractant with a hand-held blender. The extraction ratio varied from 1:3 to 1:5 (g fresh weight: ml extractant), according to the water content of the plant material. Two extractants were compared: (A) 0.2 M succinate (Na^+^), pH 5.5, containing 10 mM CaCl_2_ and (B) 1 M NaCl dissolved in buffer A; both containing polyvinylpolypyrrolidone (PVPP; 2% w/v). The parsley XET subsequently used in xenobiotics testing was extracted (from shoots of the herb, purchased at a supermarket) with buffer A without 10 mM CaCl_2_. The homogenate was stirred slowly with a magnetic stirrer for 3 h at 4 °C. After filtration through two layers of Miracloth (Calbiochem), the extract was centrifuged at 12,000*g* for 45 min; the employed supernatant had a protein concentration of 0.31 mg/ml. All enzyme extracts were stored at −80 °C. The lucerne (alfalfa; *Medicago sativa*) enzyme preparation used in Table 3 was extracted in the same way from 3-d-old seedlings, soil-grown at 25 °C.

### 5.2 Enzyme assay reagents

Tamarind seed xyloglucan (donor substrate) was a generous gift from Dr. K. Yamatoya, Dainippon Pharmaceutical Co., Japan (http://www.ds-pharma.com/). XGO–SR was prepared as described by Miller et al. (2007), starting from a mixture containing mainly XLLG, XXLG and XXXG. Reductively tritiated oligosaccharides {[1-^3^H]XXXGol, 85 MBq/μmol; (1→4)-β-d-[1-^3^H]Gal_12_-ol, 390 MBq/μmol; (1→4)-β-d-[1-^3^H]Xyl_6_-ol, 780 MBq/μmol; (1→4)--d-[1-^3^H]GalA_18_-ol, 780 MBq/μmol; (1→4)-β-d-[1-^3^H]Glc_6_-ol, 780 MBq/μmol; (1→3)-β-d-[1-^3^H]Gal_6_-ol, 780 MBq/μmol; (1→4)-β-d-[1-^3^H]Man_6_-ol, 780 MBq/μmol} and tritiated reducing oligosaccharides {[*Fuc*-1-^3^H]XXFG, 4.2 MBq/μmol; [*Gal*-6-^3^H]XLLG, 0.84 MBq/μmol} were from Edipos, The University of Edinburgh, UK (http://fry.bio.ed.ac.uk//edipos.html).

### 5.3 Xenobiotic collections

The EDI collection comprised a diverse range of 340 compounds, mainly sourced from Sigma–Aldrich (http://www.sigmaaldrich.com), the former British Drug Houses Ltd (BDH, Poole, Dorset) and Toronto Research Chemicals Inc. (TRC) (http://www.trc-canada.com). Where possible, the xenobiotics were dissolved in dimethylsulphoxide (DMSO) at 20 mg/ml; a full list, noting the exceptions to this concentration, is given in Table S2.

The ‘LATCA’ [Library of AcTive Compounds on Arabidopsis] collection is an array of 3650 compounds largely selected by virtue of bioactivity in phenotype-based screens of a number of compound libraries. Approximately 1200 compounds in the library had been shown to cause at least 20% growth inhibition in etiolated *Arabidopsis* hypocotyls at 25 μM in screens of the 10,000-member DiverSet library (Zhao et al., 2007) from Chembridge Corporation (CA, USA), the 1280-member Library Of Pharmacologically Active Compounds (LOPAC) from Sigma–Aldrich (MO, USA) or the 2000-member Spectrum library from Microsource Discovery Systems (CT, USA). Approximately 1600 compounds had been identified as inhibitors of *Saccharomyces cerevisiae* growth in a screen of a 50,000-member diversity library from Maybridge Corporation (UK). The remaining compounds in the library were assembled from in-house compounds purchased from several vendors. Further details on the LATCA library can be found at http://cutlerlab.blogspot.co.uk/2008/05/latca.html and the library’s contents are available on request from SRC. LATCA compounds were obtained as 2.5 mM stocks in DMSO.

Both the LATCA and EDI collections were stored in 96-well plates at −80 °C.

For inhibitor screening, 53 new 96-well plates were set up with 50 μl of parsley enzyme extract per well, and 1 μl of DMSO-solubilised xenobiotic was added to each well, giving concentrations of 0.4 mg/ml (EDI collection, with exceptions noted in Table S2) or 50 μM (LATCA collection) and 2% v/v DMSO. Given the typical molecular weights of these xenobiotics, this implies that the EDI compounds were tested at a *ca.* 10–40-fold higher concentration than the LATCA compounds.

### 5.4 Dot blot XET assays

XET test-paper was prepared as before (Fry, 1997) and 112 × 75-mm sheets were attached to cellulose acetate overhead-projector sheets with ‘Spraymount’ adhesive. Each sheet was placed on a pre-cooled glass plate in a cold room at 4 °C.

For the phylogenetic survey (Fig. 1), 4 μl of plant extract was applied to each of the 96 positions on one sheet quickly enough to avoid drying. The paper was covered with a second sheet of acetate so that humid conditions would be maintained, sealed with masking tape and incubated in a polythene bag surrounded by wet paper towels. The set-up was incubated under a heavy weight in the dark at 20 °C for 0.5–13 h. The paper was then washed for 1 h in formic acid/ethanol/water (1:1:1 by vol.) and dried, and the fluorescent spots were photographed under a 254-nm ultraviolet lamp by means of a Doc-It Imaging System.

For inhibitor screening (Fig. 2), 4 μl of a parsley enzyme/xenobiotic mixture was applied to each position on a sheet. Incubation at 20 °C for 2 h, washing, drying and photography were conducted essentially as above.

### 5.5 Radiochemical XET assays

For the radiochemical re-testing of putative xenobiotic hits, each parsley enzyme/xenobiotic mixture (5 μl; 1.5 μg protein; see §5.4) was added to 5 μl of a substrate mixture containing 0.3% tamarind xyloglucan and 1 kBq [^3^H]XXXGol to give a final reaction mixture composition of 0.15% xyloglucan, 1.2 μM [^3^H]XXXGol and 100 mM succinate (Na^+^), pH 5.5, and incubated at 20 °C for 2 h. Thus, for the radiochemical re-screening, compounds from the EDI and LATCA collections were present in the reaction mixture at final concentrations of approximately 200 μg/ml and 25 μM respectively. After 2 h, the reaction stopped with 10 μl 90% formic acid, and the products were dried onto Whatman No. 3 paper. The paper was washed overnight in running water, re-dried and assayed for bound ^3^H by scintillation counting.

Sixteen of the xenobiotics that exhibited strong inhibitory effects at 200 μg/ml were further tested at lower concentrations. The parsley enzyme concentration was held constant, and the xenobiotics were tested at 200, 160, 120, 80, 40, 20, 10 and 0 μg/ml (concentrations in the final reaction mixture). Other details were as above.

To test the influence of light on the inhibitory effect of riboflavin, we repeated the radiochemical assay as above, with or without riboflavin [200 μg/ml (=530 μM) in the reaction mixture], at 20 °C for 2 h, either under normal bright laboratory lighting (fluorescent lamps; 1100 lux) or in a darkened room (0.2 lux). The riboflavin-free controls received the same DMSO concentration.

### 5.6 Dot blot assays for nine additional wall enzyme activities

We devised dot-blot methods for assaying nine additional wall enzyme activities, for example exo- and/or endo-enzyme activities that hydrolyse the pectic domain (1 → 4)-β-d-galactan (β-galactosidase and/or β-galactanase) and the hemicellulosic backbone (1 → 4)-β-d-xylan (β-xylosidase and/or β-xylanase).

Radiolabelled oligosaccharide ([1-^3^H]Gal_12_-ol or [1-^3^H]Xyl_6_-ol; 1 kBq; containing a trace of Orange G) was dried into each well of a 96-well plate. Parsley enzyme extract (5 μl) containing a specific xenobiotic [routinely 0.4 mg/ml final concentration for the LAB collection, or 50 μM (≈0.01–0.04 mg/ml; see §5.3) for the LATCA collection] was then added, which dissolved the ^3^H-oligosaccharide and Orange G (checked visually). The mixture was incubated for 24 h (for Gal_12_-ol) or 48 h (for Xyl_6_-ol), then the 5-μl reaction mixture was dried onto each position on a piece of plain Whatman No. 1 paper in 96-well format. Visual inspection of the Orange G verified that the samples had been correctly applied to the paper. The dried paper was washed with 70% ethanol (for Gal_12_-ol) or 60% ethanol (for Xyl_6_-ol), which removed any enzyme-generated low-M_r_ hydrolysis products (and the Orange G), then the paper was fluorographed, revealing any remaining ^3^H-oligosaccharides that were too big to dissolve in the selected ethanol concentration. Thus, in this assay, radioactive spots reveal inhibition of the enzyme.

Comparable ‘^3^H/paper/ethanol’ assays were also developed for the following seven additional wall enzyme activities extracted from lucerne (*Medicago*) seedlings (with the radioactive substrate, incubation time and ethanol concentration indicated in parentheses): α-l-fucosidase ([*Fuc*-1-^3^H]XXFG, 24 h, 96%); (1→4)-α-d-galacturonidase + endo-polygalacturonidase (reductively tritiated galacturonooctadecaose, i.e. (1→4)-α-d-GalA_17_-l-[6-^3^H]galactonate, 96 h, 70%); α-d-xylosidase + β-d-glucosidase ([1-^3^H]XXXGol, 96 h, 80%); (1→4)-β-d-glucosidase ([1-^3^H]cellohexaitol, 24 h, 40%); β-d-galactosidase ([*Gal*-6-^3^H]XLLG, 12 h, 90%); (1→4)-β-d-mannosidase ([1-^3^H]mannohexaitol, 24 h, 70%); and (1→3)-β-d-glucosidase ([1-^3^H]laminarihexaitol, 1 h, 90%).

### 5.7 Assaying the effects of selected xenobiotics on cell-suspension cultures

Cell-suspension cultures of “Paul’s Scarlet” rose [a complex hybrid; genus *Rosa*; isolated in October 1957 from sections of young stem material; Nickell and Tulecke, 1959) and maize (*Zea mays* L., Black Mexican Sweetcorn, donated by Dr. I. Moore, Department of Botany, University of Edinburgh) were grown under constant dim illumination (10 μmol m^−2^ s^−1^) on an orbital shaker at 25 °C. Rose cells were maintained as described by Chormova et al. (2014) and maize cells as described by Kerr and Fry (2003).

Eight xenobiotics that inhibited XET activity were selected for biological testing. Mini-cultures [2 ml, 5% (v/v) SCV; transferred from a standard culture 7 d after sub-culturing] were incubated in 12-well tissue culture microplates with the lids closed loosely. Xenobiotics were added as concentrated stocks in DMSO (final DMSO concentration 2%), and incubation was continued aseptically under standard conditions of temperature, lighting and shaking. After 4 d, 1 kBq of [^14^C]proline was added per mini-culture. A small volume of medium (50 μl) was sampled 2 and 4 h after [^14^C]proline addition and assayed for remaining extracellular ^14^C. The culture was brought up to 10 ml with water and the settled and packed cell volumes (SCV, PCV) were then measured (Fry and Street, 1980). Most of the culture medium was then removed and the cells were finally killed by addition of 0.5 ml of 6% SDS. After 0.5 h at 100 °C, a 100-μl aliquot of the centrifuged SDS-extract was dried onto Whatman No. 3 paper, which was then washed in 75% ethanol supplemented with 0.5% acetic acid, thus removing free [^14^C]proline and its low-M_r_ metabolites if any; radioactivity remaining on the paper ([^14^C]protein) was then assayed.

## Appendix A Supplementary data

Supplementary data This file contains supplementary figures.
