# Supplementary material for: Discovery of small molecule inhibitors of xyloglucan endotransglucosylase (XET) activity by high-throughput screening
Source: Phytochemistry. 2015 Sep;117:220–36. doi: 10.1016/j.phytochem.2015.06.016 (PMC4560162; doi:10.1016/j.phytochem.2015.06.016)
Supplement: Supplementary data — This file contains supplementary figures. [file mmc1.pptx]

## Slide 1
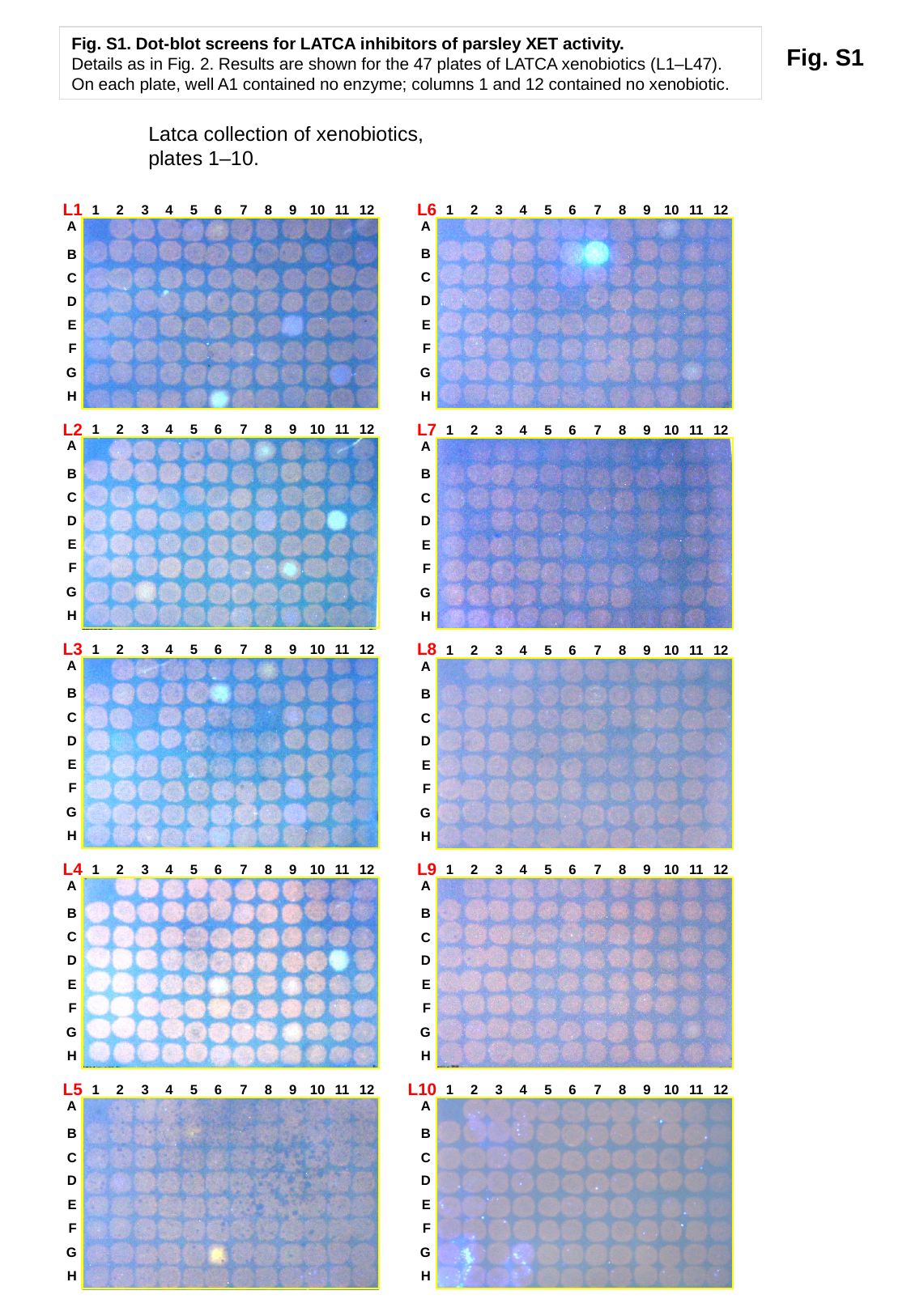

Fig. S1. Dot-blot screens for LATCA inhibitors of parsley XET activity.
Details as in Fig. 2. Results are shown for the 47 plates of LATCA xenobiotics (L1–L47). On each plate, well A1 contained no enzyme; columns 1 and 12 contained no xenobiotic.
Fig. S1
Latca collection of xenobiotics,
plates 1–10.
L1
L6
1
2
3
4
5
6
7
8
9
10
11
12
A
B
C
D
E
F
G
H
1
2
3
4
5
6
7
8
9
10
11
12
A
B
C
D
E
F
G
H
L2
L7
1
2
3
4
5
6
7
8
9
10
11
12
A
B
C
D
E
F
G
H
1
2
3
4
5
6
7
8
9
10
11
12
A
B
C
D
E
F
G
H
L3
L8
1
2
3
4
5
6
7
8
9
10
11
12
A
B
C
D
E
F
G
H
1
2
3
4
5
6
7
8
9
10
11
12
A
B
C
D
E
F
G
H
L4
L9
1
2
3
4
5
6
7
8
9
10
11
12
A
B
C
D
E
F
G
H
1
2
3
4
5
6
7
8
9
10
11
12
A
B
C
D
E
F
G
H
L5
L10
1
2
3
4
5
6
7
8
9
10
11
12
A
B
C
D
E
F
G
H
1
2
3
4
5
6
7
8
9
10
11
12
A
B
C
D
E
F
G
H

## Slide 2
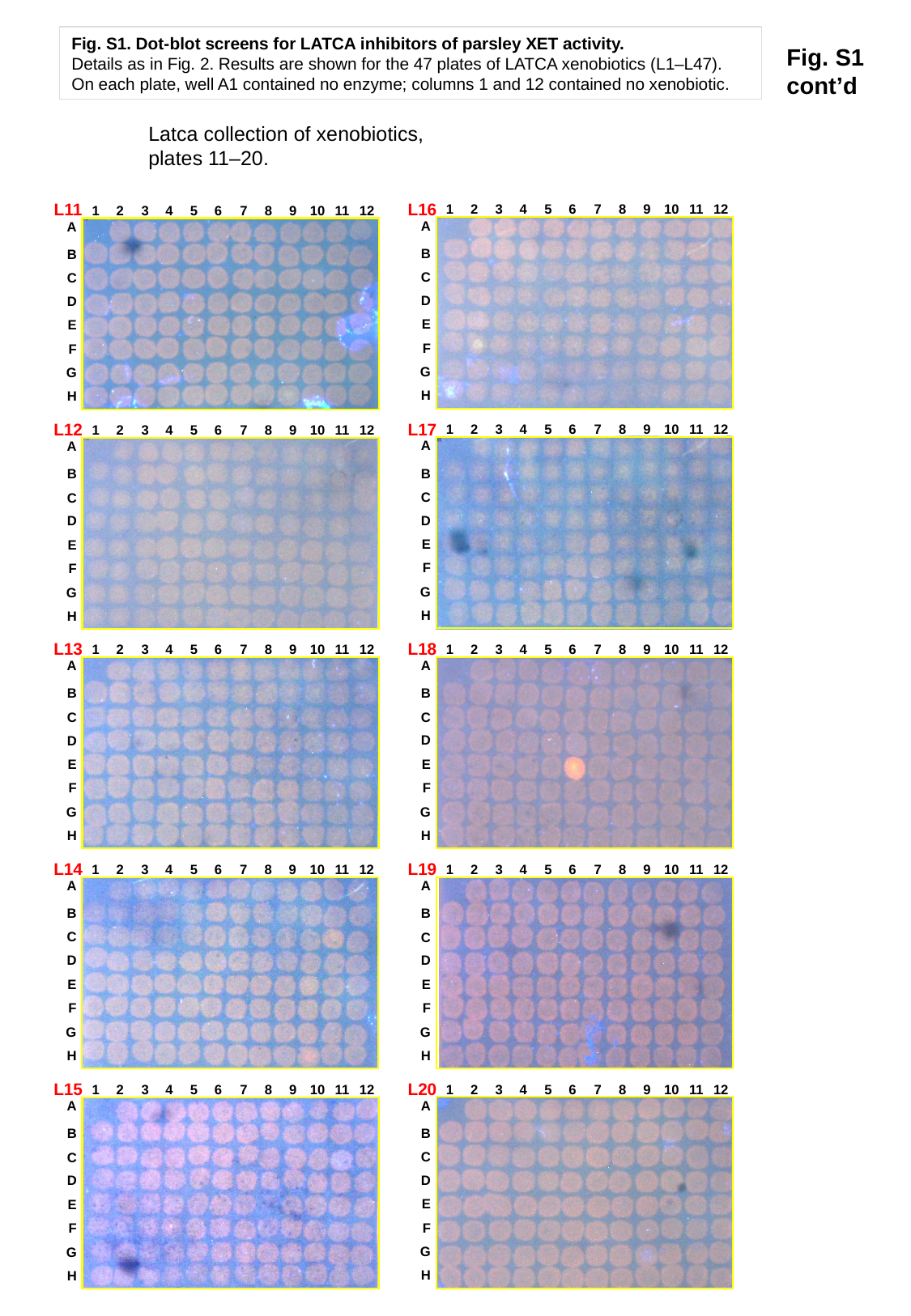

Fig. S1. Dot-blot screens for LATCA inhibitors of parsley XET activity.
Details as in Fig. 2. Results are shown for the 47 plates of LATCA xenobiotics (L1–L47). On each plate, well A1 contained no enzyme; columns 1 and 12 contained no xenobiotic.
Fig. S1
cont’d
Latca collection of xenobiotics,
plates 11–20.
L11
L16
1
2
3
4
5
6
7
8
9
10
11
12
A
B
C
D
E
F
G
H
1
2
3
4
5
6
7
8
9
10
11
12
A
B
C
D
E
F
G
H
L12
L17
1
2
3
4
5
6
7
8
9
10
11
12
A
B
C
D
E
F
G
H
1
2
3
4
5
6
7
8
9
10
11
12
A
B
C
D
E
F
G
H
L13
L18
1
2
3
4
5
6
7
8
9
10
11
12
A
B
C
D
E
F
G
H
1
2
3
4
5
6
7
8
9
10
11
12
A
B
C
D
E
F
G
H
L14
L19
1
2
3
4
5
6
7
8
9
10
11
12
A
B
C
D
E
F
G
H
1
2
3
4
5
6
7
8
9
10
11
12
A
B
C
D
E
F
G
H
L15
L20
1
2
3
4
5
6
7
8
9
10
11
12
A
B
C
D
E
F
G
H
1
2
3
4
5
6
7
8
9
10
11
12
A
B
C
D
E
F
G
H

## Slide 3
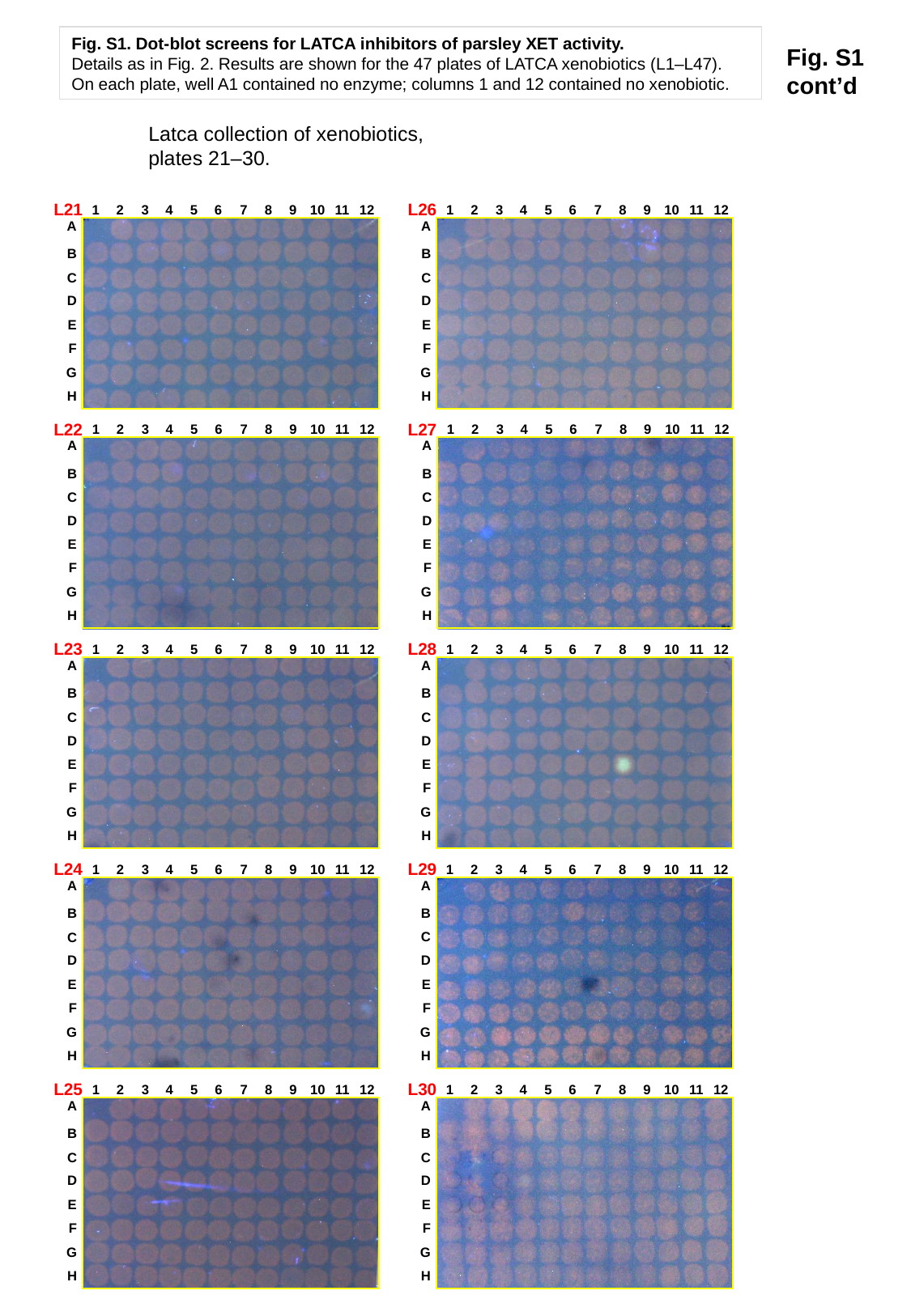

Fig. S1. Dot-blot screens for LATCA inhibitors of parsley XET activity.
Details as in Fig. 2. Results are shown for the 47 plates of LATCA xenobiotics (L1–L47). On each plate, well A1 contained no enzyme; columns 1 and 12 contained no xenobiotic.
Fig. S1
cont’d
Latca collection of xenobiotics,
plates 21–30.
L21
L26
1
2
3
4
5
6
7
8
9
10
11
12
A
B
C
D
E
F
G
H
1
2
3
4
5
6
7
8
9
10
11
12
A
B
C
D
E
F
G
H
L22
L27
1
2
3
4
5
6
7
8
9
10
11
12
A
B
C
D
E
F
G
H
1
2
3
4
5
6
7
8
9
10
11
12
A
B
C
D
E
F
G
H
L23
L28
1
2
3
4
5
6
7
8
9
10
11
12
A
B
C
D
E
F
G
H
1
2
3
4
5
6
7
8
9
10
11
12
A
B
C
D
E
F
G
H
L24
L29
1
2
3
4
5
6
7
8
9
10
11
12
A
B
C
D
E
F
G
H
1
2
3
4
5
6
7
8
9
10
11
12
A
B
C
D
E
F
G
H
L25
L30
1
2
3
4
5
6
7
8
9
10
11
12
A
B
C
D
E
F
G
H
1
2
3
4
5
6
7
8
9
10
11
12
A
B
C
D
E
F
G
H

## Slide 4
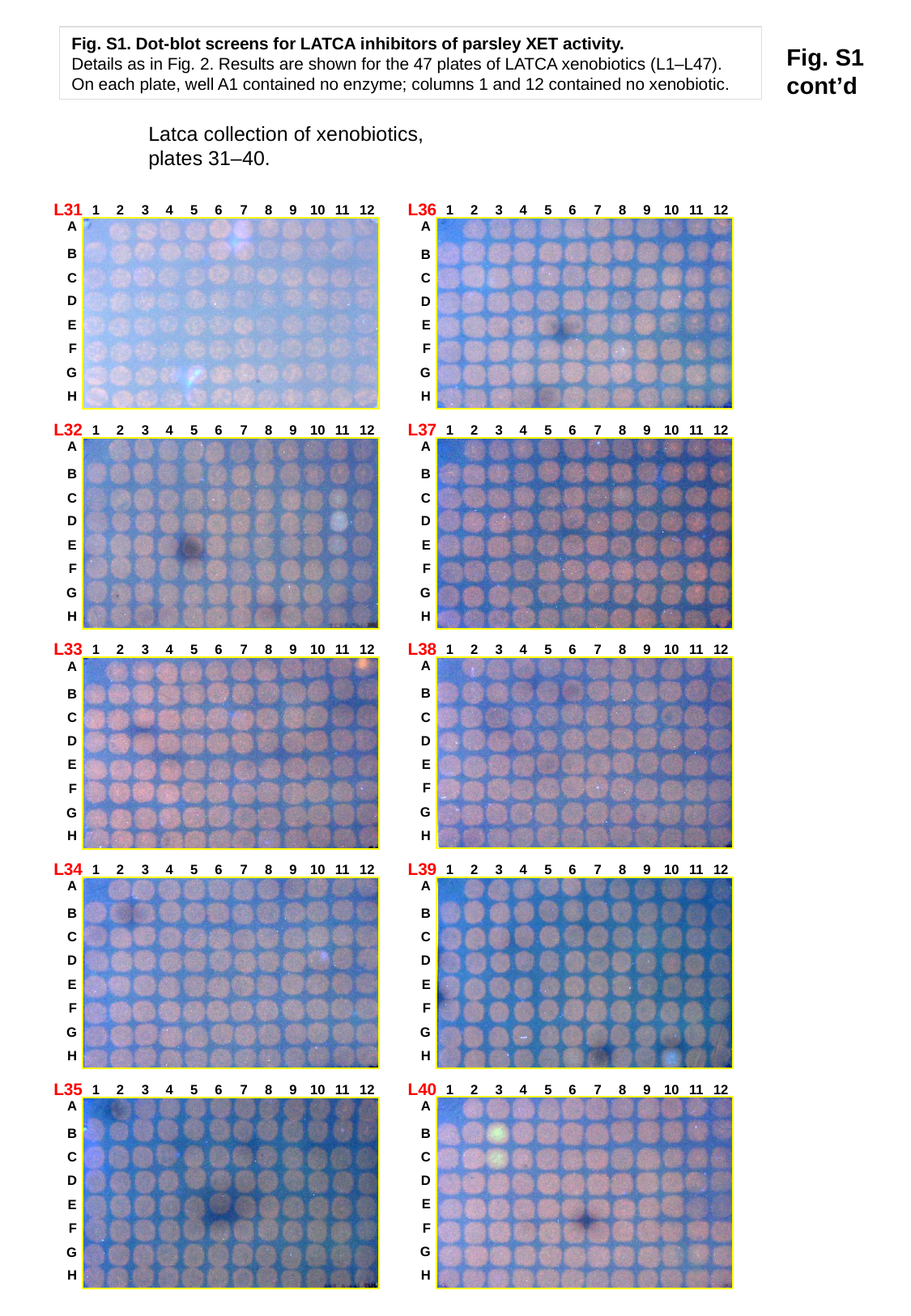

Fig. S1. Dot-blot screens for LATCA inhibitors of parsley XET activity.
Details as in Fig. 2. Results are shown for the 47 plates of LATCA xenobiotics (L1–L47). On each plate, well A1 contained no enzyme; columns 1 and 12 contained no xenobiotic.
Fig. S1
cont’d
Latca collection of xenobiotics,
plates 31–40.
L31
L36
1
2
3
4
5
6
7
8
9
10
11
12
A
B
C
D
E
F
G
H
1
2
3
4
5
6
7
8
9
10
11
12
A
B
C
D
E
F
G
H
L32
L37
1
2
3
4
5
6
7
8
9
10
11
12
A
B
C
D
E
F
G
H
1
2
3
4
5
6
7
8
9
10
11
12
A
B
C
D
E
F
G
H
L33
L38
1
2
3
4
5
6
7
8
9
10
11
12
A
B
C
D
E
F
G
H
1
2
3
4
5
6
7
8
9
10
11
12
A
B
C
D
E
F
G
H
L34
L39
1
2
3
4
5
6
7
8
9
10
11
12
A
B
C
D
E
F
G
H
1
2
3
4
5
6
7
8
9
10
11
12
A
B
C
D
E
F
G
H
L35
L40
1
2
3
4
5
6
7
8
9
10
11
12
A
B
C
D
E
F
G
H
1
2
3
4
5
6
7
8
9
10
11
12
A
B
C
D
E
F
G
H

## Slide 5
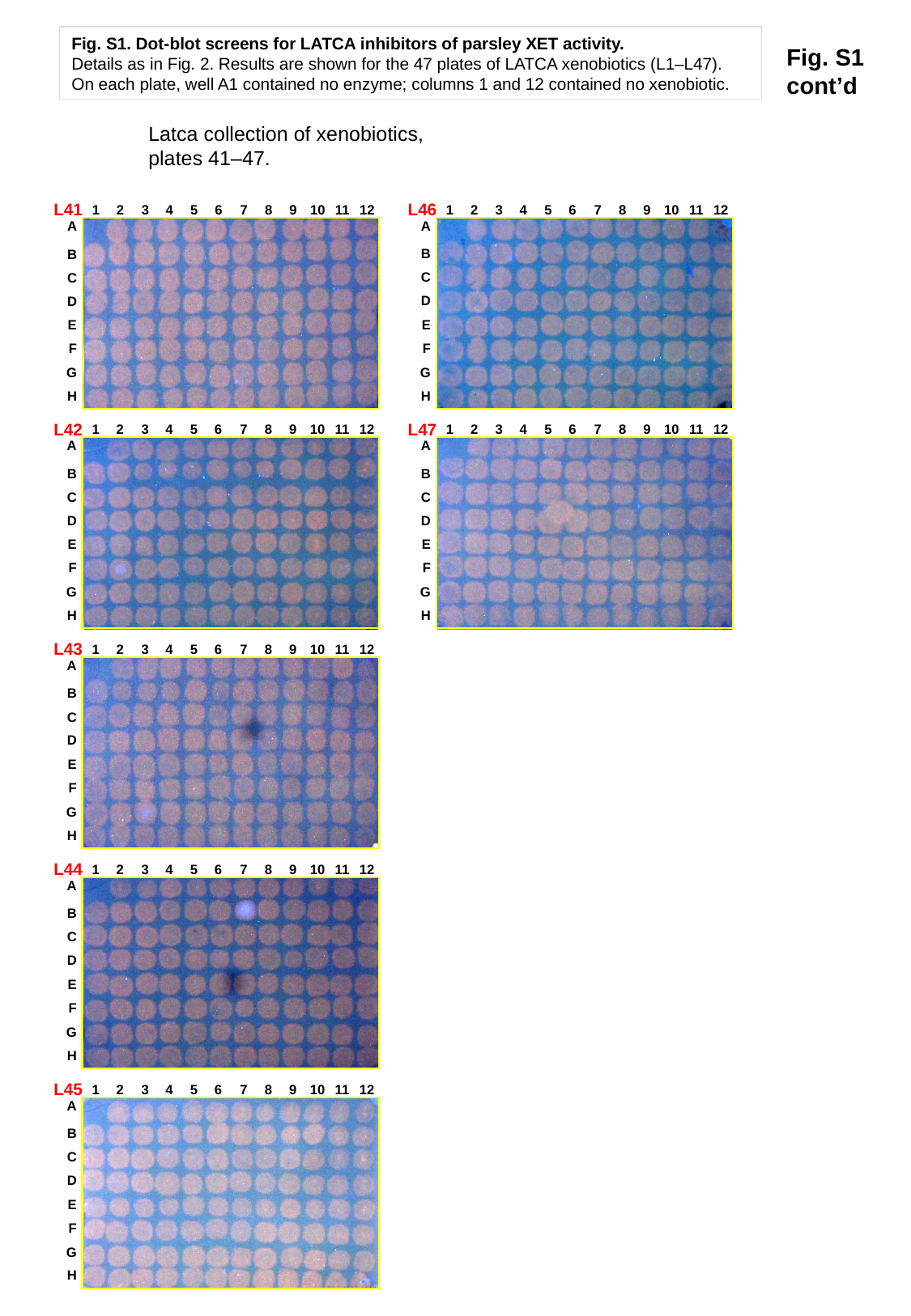

Fig. S1. Dot-blot screens for LATCA inhibitors of parsley XET activity.
Details as in Fig. 2. Results are shown for the 47 plates of LATCA xenobiotics (L1–L47). On each plate, well A1 contained no enzyme; columns 1 and 12 contained no xenobiotic.
Fig. S1
cont’d
Latca collection of xenobiotics,
plates 41–47.
L41
L46
1
2
3
4
5
6
7
8
9
10
11
12
A
B
C
D
E
F
G
H
1
2
3
4
5
6
7
8
9
10
11
12
A
B
C
D
E
F
G
H
L42
L47
1
2
3
4
5
6
7
8
9
10
11
12
A
B
C
D
E
F
G
H
1
2
3
4
5
6
7
8
9
10
11
12
A
B
C
D
E
F
G
H
L43
1
2
3
4
5
6
7
8
9
10
11
12
A
B
C
D
E
F
G
H
L44
1
2
3
4
5
6
7
8
9
10
11
12
A
B
C
D
E
F
G
H
L45
1
2
3
4
5
6
7
8
9
10
11
12
A
B
C
D
E
F
G
H

## Slide 6
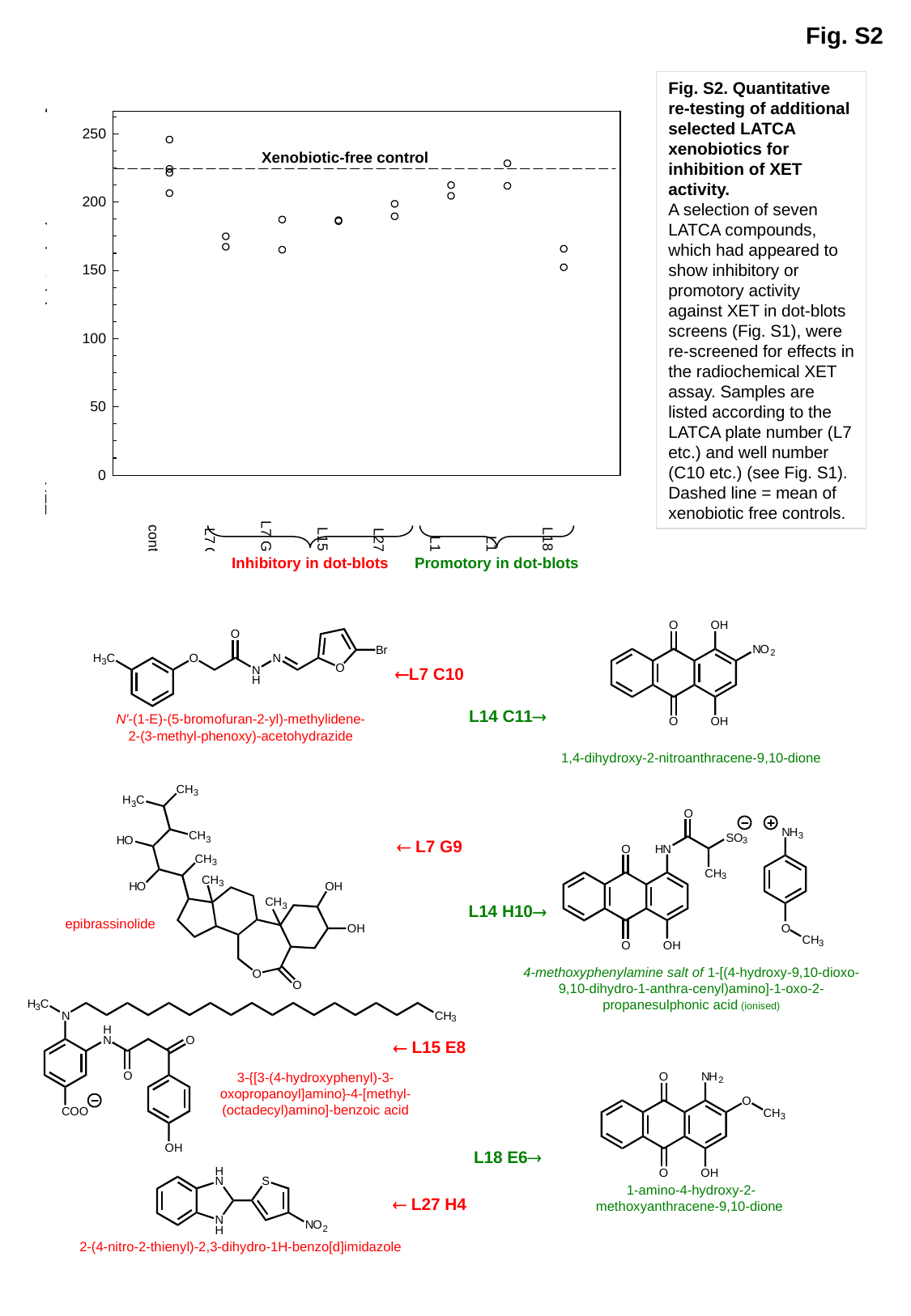

Fig. S2
Fig. S2. Quantitative re-testing of additional selected LATCA xenobiotics for inhibition of XET activity.
A selection of seven LATCA compounds, which had appeared to show inhibitory or promotory activity against XET in dot-blots screens (Fig. S1), were re-screened for effects in the radiochemical XET assay. Samples are listed according to the LATCA plate number (L7 etc.) and well number (C10 etc.) (see Fig. S1). Dashed line = mean of xenobiotic free controls.
Xenobiotic-free control
Inhibitory in dot-blots
Promotory in dot-blots
L7 C10
L14 C11
N'-(1-E)-(5-bromofuran-2-yl)-methylidene-2-(3-methyl-phenoxy)-acetohydrazide
1,4-dihydroxy-2-nitroanthracene-9,10-dione
 L7 G9
L14 H10
epibrassinolide
4-methoxyphenylamine salt of 1-[(4-hydroxy-9,10-dioxo-9,10-dihydro-1-anthra-cenyl)amino]-1-oxo-2-propanesulphonic acid (ionised)
 L15 E8
3-{[3-(4-hydroxyphenyl)-3-oxopropanoyl]amino}-4-[methyl-(octadecyl)amino]-benzoic acid
L18 E6
1-amino-4-hydroxy-2-methoxyanthracene-9,10-dione
 L27 H4
2-(4-nitro-2-thienyl)-2,3-dihydro-1H-benzo[d]imidazole
